# On-Surface Synthesis of a Large-Scale 2D MOF with Competing π–d Ferromagnetic/Antiferromagnetic Order

**DOI:** 10.1021/jacs.4c17993

**Published:** 2025-05-30

**Authors:** Federico Frezza, Manish Kumar, Ana Sánchez-Grande, Diego Soler-Polo, Manuel Carrera, Oleksandr Stetsovych, Pingo Mutombo, David Curiel, Pavel Jelínek

**Affiliations:** † 86889 Institute of Physics of Czech Academy of Sciences, Cukrovarnická 10, 16200 Prague 6, Czech Republic; ‡ Faculty of Nuclear Sciences and Physical Engineering, Czech Technical University in Prague, Břehová 78/7, 11519 Prague 1, Czech Republic; § Department of Condensed Matter Physics, Faculty of Mathematics and Physics, Charles University, CZ12116 Prague 2, Czech Republic; ∥ Department of Organic Chemistry, 16751 University of Murcia Campus of Espinardo, 30100 Murcia, Spain; ⊥ Département de Raffinage et Pétrochimie, Faculté de Pétrole, Gaz et Énergies Renouvelables, Université de Kinshasa, BP 127, Kinshasa XI, République Démocratique du Congo; # CATRIN-RCPTM, Palacký University, Šlechtitelů 27, 783 71 Olomouc, Czech Republic

## Abstract

Metal–organic frameworks (MOFs) represent an interesting class of versatile materials with important properties, including magnetism. However, the synthesis of atomically precise large-scale 2D MOFs with nontrivial strong magnetic coupling represents a current research challenge. In this regard, we report on the synthesis of a high-quality large-scale 2D MOF, with strong π–d magnetic exchange coupling. To this aim, we present a new two-step synthetic approach that consists of the initial formation of an extended supramolecular organic framework on a Au(111) surface, establishing the large-scale order of organic ligands and their subsequent metalation by single cobalt atoms assisted by annealing. Moreover, we show that the usage of radical asymmetric organic ligands enables us to form a magnetic 2D MOF with strong π–d electron interactions. According to the multireference calculations, the 2D MOF shows complex spin interactions beyond the traditional superexchange mechanism, with the interplay between antiferromagnetic and ferromagnetic couplings. We anticipate that this synthetic strategy can be adapted to different approaches, such as liquid interfaces or insulating substrates, to synthesize high-quality 2D MOFs. Accompanied by the high control with atomic precision over the magnetic properties of the ligands and metals, this approach enables the formation of large-scale 2D MOFs with complex spin interactions, which will open new avenues in the field of 2D magnetic materials.

## Graphical abstract

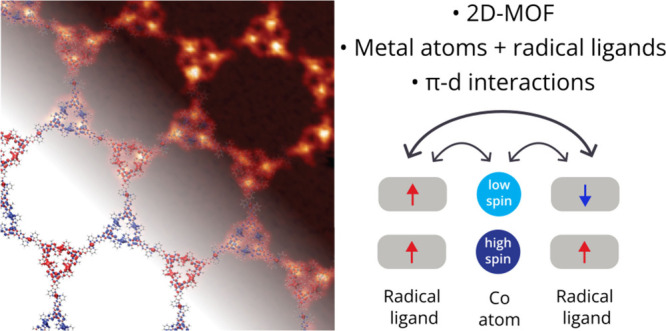

## Introduction

Metal–organic frameworks (MOFs) have revolutionized the field of materials science in the past 30 years. These reticular materials, made up of metal cations and organic ligands, exploit the formation of coordinate bonds to build up extended networks.^1–4^ The interest in these porous materials comes from the versatile combination of organic ligands and metal centers, forming different coordination spheres and providing a straightforward methodology toward an infinite variety of largely ordered two- or three-dimensional (2D, 3D) systems. In particular, 2D MOFs represent unique ultrathin 2D materials with accessible pores and high surface area, presenting an interest in catalysis, drug delivery, sensing, or energy storage.^5–8^ They also represent an ideal platform for realizing 2D magnets.^9–18^ Several examples of 2D MOFs with ferromagnetic^19,20^ and antiferromagnetic order^21^ were reported. Within this context, the traditional approach relies on magnetic metallic centers coordinated to organic ligands (diamagnetic) whose design determines the spin coupling between the magnetic moments of the metal centers.^22^ The magnetic coupling through the bridging ligand results in a more predictable antiferromagnetic coupling (Figure 1a, left panel).^23^ Nevertheless, this approach presents some limitations, such as the mandatory usage of short linkers to enable the exchange interaction between metal centers. This substantially limits the porosity of 2D MOFs and their applications. In many cases, the exchange interaction between metal centers mediated by diamagnetic ligands implies the absence of superexchange interaction accompanied by low Curie temperatures.^9^

**Figure 1:**
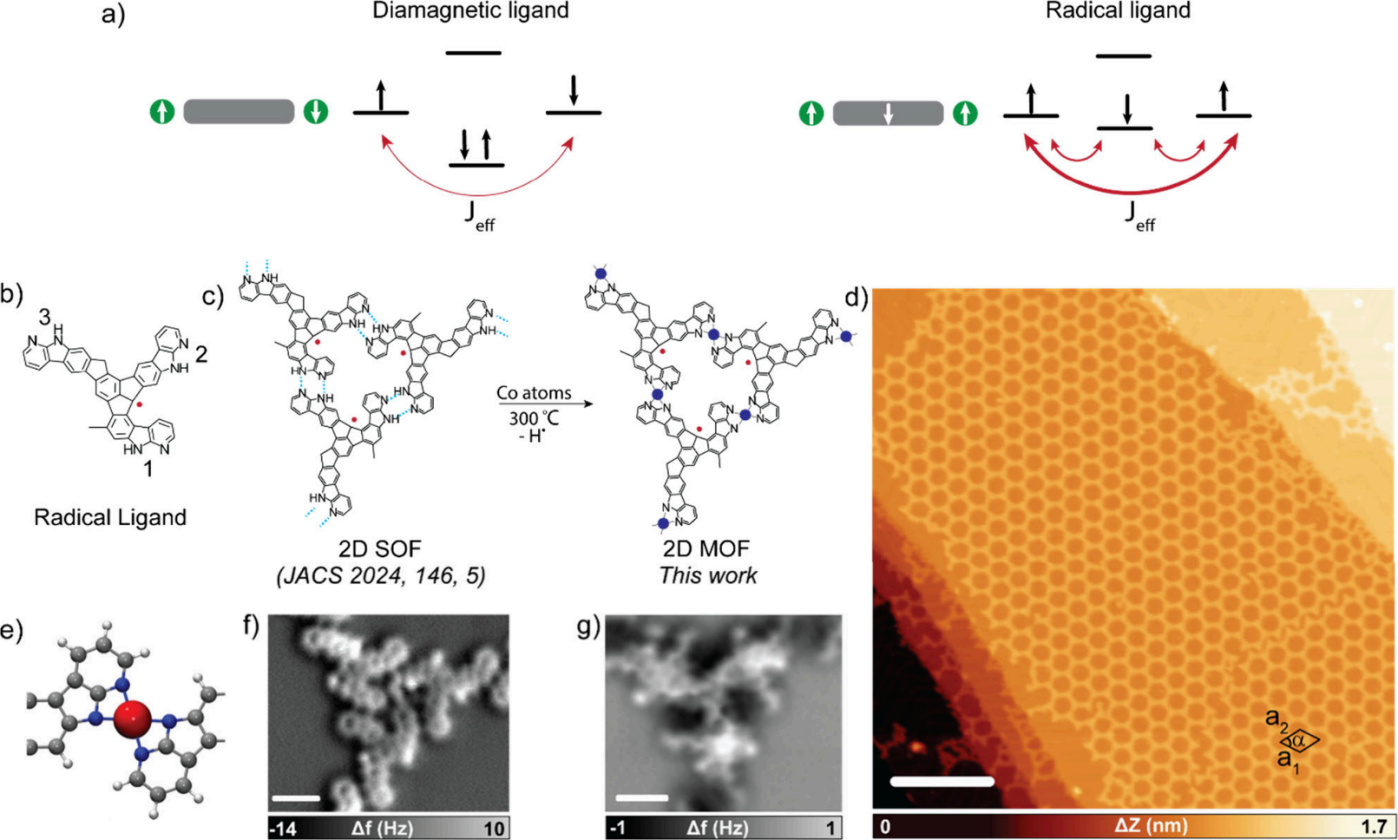
On-surface synthesis of radical-as-ligand containing MOFs on Au(111). (a) Schematic representation of the superexchange mechanism and radical-mediated exchange coupling. Metal atoms and organic ligands are represented with green circles and grey rectangles, respectively. (b) Chemical structure of the radical ligand. (c) Chemical route from the 2D supramolecular framework to the 2D metal–organic framework on Au(111). (d) Constant-current overview STM image of the cobalt-coordinated 2D MOF containing organic radical ligands (*V*_b_ = 100 mV, *I*_t_ = 10 pA and scale bar = 28 nm). (e) Ball-and-stick model showing the top view of the cobalt coordination between the 7-azaindole units. (f) Nc-AFM image of a trimer from the 2D SOF on Au(111) (*V*_b_ = 1 mV and scale bar = 0.9 nm). (g) Nc-AFM image of a trimer after Co coordination forming the 2D MOF on Au(111) (*V*_b_ = 1 mV and scale bar = 0.8 nm).

One alternative methodology investigated in bulk MOFs uses organic radicals as ligands, thus facilitating the magnetic coupling between the metal centers incorporating spin carriers into the ligands.^24–26^ “Radical-as-ligand” strategies lead to a more complex electronic picture (Figure 1a, right panel), giving rise to several low-lying energy states, each characterized by different electronic configurations and spin multiplicities. The resulting magnetic ground state is sensitive to different parameters, such as the geometry^27^ or the chemical bonding.^28–30^ The magnetic interplay between π- and d-radicals results in complex spin interactions, as demonstrated at the atomic scale for metalated porphyrins presenting both d- and π-electrons^31^ and recently for coordination complexes employing open-shell molecular systems.^32,33^ Thus, using organic radical ligands opens the possibility of exploiting the π–d interactions on a large scale. Nevertheless, the main obstacle to the radical-as-ligand approach resides in the low stability of organic radicals, which makes their synthesis difficult and precludes the formation of high-quality materials.

In addition, the stable long-range magnetic arrangement of low-dimensional materials is very susceptible to atomic defects, requiring the synthesis of high-quality 2D MOFs.^34^ Despite the large number of 2D MOFs reported, limitations are found in growing atomically precise large-scale 2D MOFs.^5,35^ Typically, their synthesis is based on bottom-up nucleation and growth processes, which might result in cluster formation, metastable intermediate phases, limited solubility, and competition with vertical growth, limiting the quality and control of the thickness of the 2D MOFs. Another common approach is top-down exfoliation. However, restacking is a common limitation due to the instability of the nanosheets. Therefore, the synthesis of high-quality atomically precise large-scale 2D MOFs with tailored properties, such as magnetic functionalities, calls for new synthetic methods. (the stepwise process is illustrated in Scheme 1)

In this work, we present a new two-step synthetic methodology to grow large-scale, atomically precise magnetic 2D MOFs. We combine supramolecular chemistry, on-surface synthesis, and coordination chemistry to fabricate a high-quality 2D MOF featuring intricate magnetic functionalities. The first step consists of organizing the organic ligands, forming large-scale 2D supramolecular frameworks, becoming an ideal template for MOF formation. The second step involves selective metalation at the chelating sites, assisted by annealing, to synthesize atomically precise, large-scale magnetic 2D MOFs on metallic surfaces, where the quality and topology of the template (supramolecular framework) are preserved. Moreover, we demonstrate that this synthetic approach can also incorporate the “radical-as-ligand” concept, thanks to the ultrahigh vacuum on-surface synthesis that allows the formation and stabilization of radical ligands.^36^ This new synthetic protocol enables us to form large-scale 2D magnetic MOFs with a complex spin texture system dominated by different π–d interactions. Scanning tunneling microscopy/spectroscopy (STM/STS) measurements corroborated by theoretical calculations confirm the magnetic ground state with strong π–d coupling. Multireference calculations as well as density functional theory (DFT) calculations predict complex long-range magnetic order with low and high spin Co centers and an interplay between ferromagnetic and antiferromagnetic coupling.

**Scheme 1:**
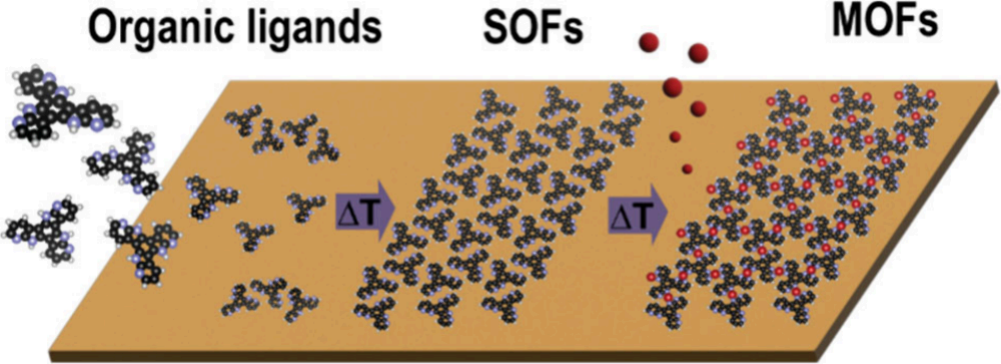
Representation of the Two-Step Synthetic Method To Grow High-Quality 2D MOFs on Surfaces^a^

a The two-step on-surface synthesis consists of the formation of long-range ordered supramolecular organic frameworks (SOFs) followed by deposition of single metal atoms and subsequent annealing providing metal–organic frameworks (MOFs).

## Results and Discussion

Our starting point is a supramolecular organic radical framework recently reported by us.^37^ The synthesis of 1,3,5-tris­(methyl-α-carbolinyl)­benzene precursor **I** sets the first step for the subsequent on-surface synthesis of tripodal π-conjugated ligands (Figure S1). Thus, the sublimation of **I** followed by annealing at 325 °C affords the formation of a radical supramolecular organic framework (SOF) consisting of monoradical ligands (see scheme in Figure S1 and Figure 1b) that integrate three 7-azaindole units to promote hydrogen bonding between molecular units. The resulting framework is constituted by a motif of homochiral trimers (see Figure 1c), which self-assemble and spread into dodecamers, forming a radical 2D SOF. The presence of the monoradical on the ligand gives rise to a Kondo resonance^38,39^ (see Figure S2a), revealing the absence of magnetic interactions between adjacent molecules connected by hydrogen bonds. Therefore, the supramolecular approach facilitates the formation of large defect-free areas of SOFs on Au(111) covering hundreds of nanometers. The mesoscale extension of the 2D SOF has been demonstrated by low-energy electron microscopy (LEEM).^40^ Moreover, the 7-azaindole unit connections establish a suitable arrangement for the formation of four-coordinate metal complexes. Consequently, the SOF preorganization represents an ideal template for the preparation of large-scale 2D magnetic MOFs with quality comparable to that of the original SOF.

Next, we deposited cobalt atoms by electron-beam deposition onto the SOF sample at room temperature (RT). The subsequent thermal activation step at 300 °C induces the removal of the hydrogen atoms from the pyrrole units of the 7-azaindole groups and the coordination of the cobalt centers, as illustrated in Figure 1c,e. The successful coordination of the cobalt atoms was confirmed by high-resolution noncontact atomic force microscopy (nc-AFM) measurements acquired with a CO-functionalized tip, with the Co-coordinated positions presenting a darker contrast in the constant-height frequency-shift images.^41^ Namely, Figure 1g reveals a significant change in the frequency shift contrast between ligands compared to the SOF; see Figure 1f and Figure S3. The lattice of the network was preserved after cobalt coordination (unit cell parameters: *a*_1_ = *a*_2_ = 6.4 nm and α = 60°), as was its quality, finding large areas almost without defects in hundreds of nanometers, as shown in Figure 1d and Figure S4, which is comparable to the quality of the SOF template. More information about the stepwise growth of the 2D MOF driven by the 2D preorganization of the ligands is provided in the experimental note in the Supporting Information.

We performed STS measurements to characterize the electronic and magnetic properties of the 2D MOF. Long-range differential conductance (d*I*/d*V*) spectra reveal a band gap of ∼3.2 eV; see Figure S5. Figure 2b displays low-energy d*I*/d*V* curves over different parts of the framework (positions depicted in Figure 2a), revealing the presence of inelastic excitation signals on the ligands and Co centers.

**Figure 2:**
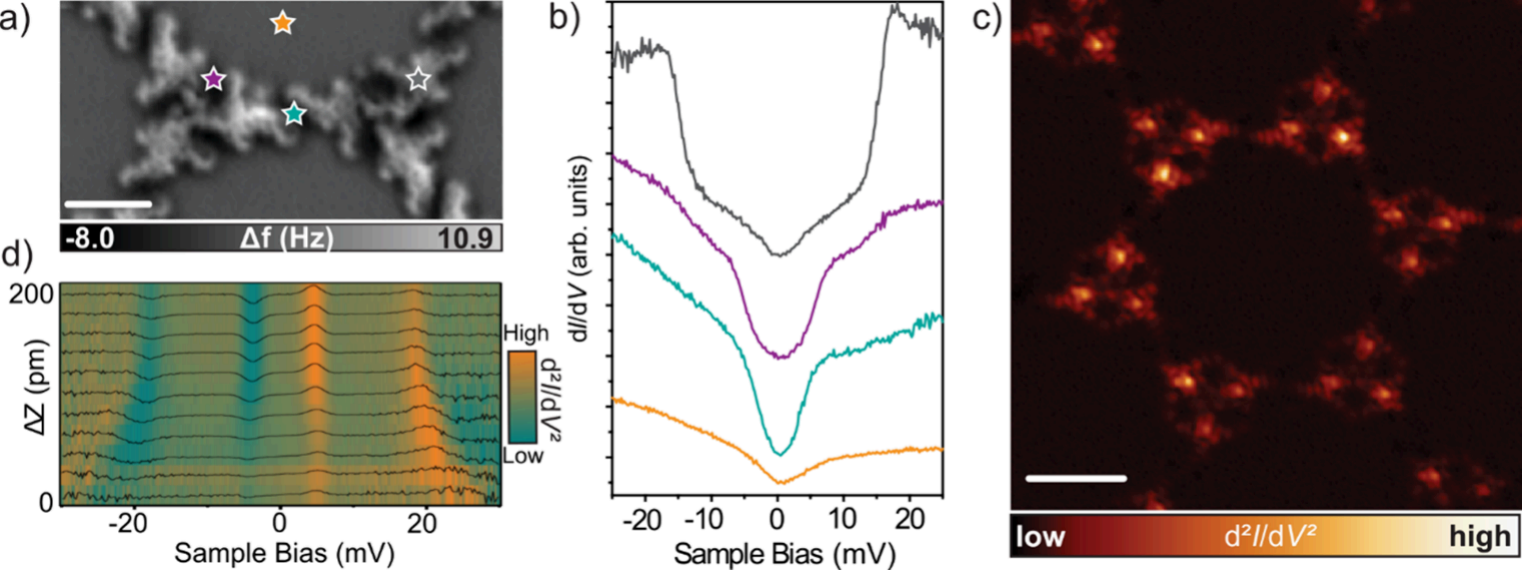
Magnetic characterization of radical-as-ligand containing MOF on Au(111). (a) Nc-AFM image of two adjacent trimers (*V*_b_ = 1 mV and scale bar = 1.6 nm). (b) Low-energy differential conductance d*I*/d*V* spectra acquired over the positions depicted in (a). (c) Experimental constant-height d^2^ *I*/d*V* ^2^ spin excitation map (*V*_b_ = 14 mV and scale bar = 2.2 nm). (d) Low-energy d^2^ *I*/d*V* ^2^ spectra plotted in a color map as a function of the tip–sample distance acquired over the organic ligands employing a nickelocene-functionalized tip. The intensities of the spectra have been normalized for easier comparison.

The spectrum acquired over the ligand (gray curve in Figure 2b) shows a V-shaped curve with two abrupt conductance steps symmetric around the Fermi energy with different slopes. We assigned this feature to spin-flip excitations, anticipating the magnetic exchange coupling between different spins on the ligands and cobalt centers. Note that this feature is consistently present throughout the network (see Figure S2b). The inelastic electron tunneling spectroscopy (IETS) measurements in Figure S6a show an effective exchange coupling of 14 meV. To confirm the magnetic origin of the signals in Figure 2b, we performed IETS measurements employing a nickelocene (Nc) functionalized tip, which has been proven to act as a magnetic sensor at the single-molecule level.^42–45^ Figure 2d shows a clear interaction of the Nc with the organic radical ligands, observing joint spin-flip excitation signals at ±18 mV that shift by approaching the tip, confirming the magnetic nature of the conductance steps presented in Figure 2b.

In the case of the Co centers, we find two different STS features, depending on their bonding configuration to the ligand. These different configurations of the Co centers are attributed to the asymmetric geometry of the ligand, with three arms labeled in Figure 1b. As shown in Figure S12, the topology of the 2D MOF imposes only two types of connections between the arms. The connection within the trimer is formed between 1 and 2 arms, while between the adjacent trimers, arms 3 face each other. Thus, these two connecting patterns give rise to two slightly different coordination environments, which causes distinct hybridization of the Co centers with ligands. For the cobalt centers within the trimer motif (violet curve in Figure 2b), in the STS we detect weak steps at ±14 meV and pronounced features at ± 5 meV. On the other hand, in the STS acquired over the cobalt center interconnecting the trimers (cyan curve in Figure 2b), we detect a feature at Fermi energy. The distinct d*I*/d*V* spectra recorded on the two cobalt centers suggest different magnetic states of the cobalt centers within the trimer and those interconnecting the trimers. Additionally, we recorded d*I/*d*V* spectra over a molecule at the edge of the framework. In this case, the spectrum presents a signal assigned to spin flip excitations at ≈6 meV (see Figure S9), whose magnetic nature was also confirmed by IETS measurements employing Nc functionalized tip.

Furthermore, we recorded d^2^ *I*/d*V* ^2^ maps with a CO-functionalized tip to demonstrate extended magnetic interactions at 14 meV. Figure 2c shows the d^2^ *I*/d*V* ^2^ map recorded at *V*_b_ = 14 mV, revealing the presence of the IETS signal on each molecular unit as well as a faint signal on metal centers (see also Figure S6b). Additionally, Figure S6b shows some “dark” molecules most likely related to the presence of a defect. However, overall, we can confirm that most of the molecules present the spin excitations signal at 14 mV. The presence of spin-excitations on both the ligand and Co centers reveals the strong magnetic coupling across the 2D MOF.

To experimentally gain more insight into the intricate magnetic interaction between spins on ligands and different Co centers, we prepared a new sample with low cobalt coverage, aiming to isolate the fundamental π–d interactions that give rise to the overall magnetic order. We followed the protocol described above but limited the number of cobalt atoms on the surface. In the case without any Co center directly coordinated with the ligand (Figure 3a,b), we observe a Kondo resonance on the ligand equivalent to that measured in the 2D SOF sample (Figure 3c). This reveals the absence of magnetic interactions between radical ligands not directly connected by Co centers. In the case of the ligands coordinated with only one Co center, we find two possible cases: the one with the Co center in the 3–3 connection (Figure 3d,e) and the second with the Co coordinated in the 1–2 connection (Figure 3g,h). In contrast to the fully coordinated MOF shown in Figure 2b characterized by a V-shaped curve, here both spectra present a typical U-shaped dip. The STS measurements over the ligands reveal two different scenarios, with the larger magnetic exchange of 8 meV for the 3–3 coupling (see Figure 3f), in contrast to the 3 meV for the 1–2 coupling (Figure 3i). These STS signals are consistent with the spectra recorded on the Co positions (Figure S10). To get more information about the impact of the chemical environment of adjacent molecules on the magnetic properties, see Figure S11. The experimental observations in the low cobalt coverage sample point out two important results. First, the quenching of the Kondo signal and emergence of spin-excitation reveal a strong π–d coupling between the d-orbitals of Co centers and π-radicals on the ligands. Second, the different values of the spin excitations for the 1–2 and 3–3 couplings indicate distinct π–d couplings, implying the presence of two inequivalent Co centers. The different magnetic interactions can be associated with the asymmetrical geometry of the ligand, which has a direct impact on the molecular orbitals. Figure S12c shows the SOMO orbital of the ligand, revealing different bonding/nonbonding characters over the 7-azaindole units. This leads to different coordination of Co centers with the ligands for 3–3 and 1–2 coupling, respectively (Supplementary Note).

**Figure 3:**
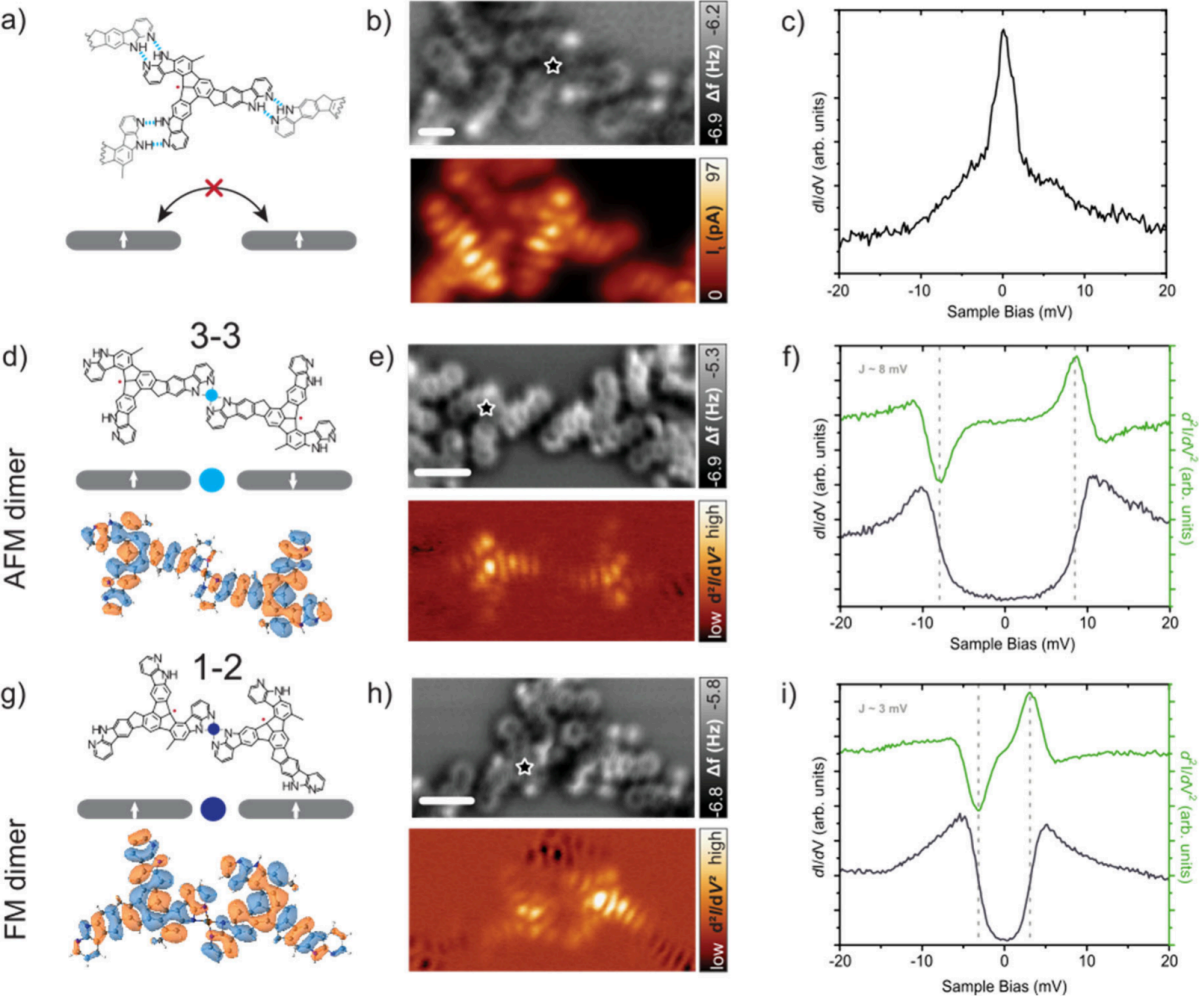
Magnetic characterization of Co-free ligand and 1–2 and 3–3 couplings. (a) Chemical sketch of a Co-free ligand. (b) nc-AFM image and constant-height STM image of Co-free ligand (*V*_b_ = 4 mV and scale bar = 0.5 nm). (c) Low-energy d*I*/d*V* spectrum acquired over the organic ligand (position depicted in panel b). (d) Chemical sketch and simulated natural transition orbital at 8 mV from doublet to quartet spin state of 3–3 coupling. Cobalt center coordinated to the 3–3 coupling is represented as a light blue circle. (e) nc-AFM image and constant-height d^2^ *I*/d*V* ^2^ spin excitation map at 8 mV of the 3–3 coupling (scale bar = 1 nm). (f) Low-energy d*I*/d*V* and d^2^ *I*/d*V* ^2^ spectra acquired over the organic ligand involved in the 3–3 coupling (position depicted in panel e). (g) Chemical sketch and simulated natural transition orbital at 4 mV from the sextet to quartet spin state of 1–2 coupling. Cobalt center coordinated to the 1–2 coupling is represented as a dark blue circle. (h) nc-AFM image and constant-height d^2^ *I*/d*V* ^2^ spin excitation map at 3 mV of the 1–2 coupling (scale bar = 1 nm). (i) Low-energy d*I*/d*V* and d^2^ *I*/d*V* ^2^ spectra acquired over the organic ligand involved in the 1–2 coupling (position depicted in panel h).

To gain a deeper understanding of the magnetic order in the 2D MOF, we carried out theoretical simulations, including total energy DFT and quantum chemistry complete active space configuration interaction (CASCI) calculations. In addition, we employed a many-body model Hamiltonian to provide more in-depth information about exchange mechanisms between localized d-orbitals and extended π-orbitals beyond superexchange scheme.

First, we carried out total energy spin-polarized DFT simulations of a free-standing 2D MOF, optimizing the atomic structure and the unit cell. We found two nonequivalent types of Co centers with low- and high-spin configurations; see Figure 4a. Namely, we found three Co centers within the trimer connected via 1–2 coupling in the high-spin state with three unpaired electrons in d-orbitals with parallel spin alignment. Figure 4b shows the optimized structure with a Co center coordinated with four N atoms with bond lengths in the range of 1.92–1.96 Å. On the contrary, the Co center connected via 3–3 coupling has a low-spin configuration with three unpaired electrons, but one of them has an opposite spin alignment. Also, the coordinated Co atom in the 3–3 coupling has slightly different bonding configurations as compare to 1-2 coupling; see Figure 4b. We also carried out DFT calculations, including the Au(111) surface, keeping the optimized unit cell of 2D MOF from the gas phase calculation; see Figure S18 (see Supplementary Note for details). The optimized structure reveals only weak interaction between 2D MOF and Au(111) surface with an average distance between top layer of Au(111) surface and Co atoms being 3.12 Å. Figure 4c displays the calculated spin density of the obtained ground state, revealing a complex ferromagnetic/antiferromagnetic order. The trimers consisting of three ligand centers coupled with three Co centers via 1–2 coupling shows ferromagnetic coupling, while the interaction mediated by Co center via 3–3 coupling imposes the antiferromagnetic coupling between two trimers. The DFT calculations indicate the crucial role of the radical ligands in facilitating the magnetic interaction across the 2D MOF while preserving its large porosity; see Figure S13. However, there is a question of whether single determinant DFT methods can provide reliable information about the electronic and magnetic structures of a 2D MOF, including both unpaired electrons in d- and π-orbitals.

**Figure 4:**
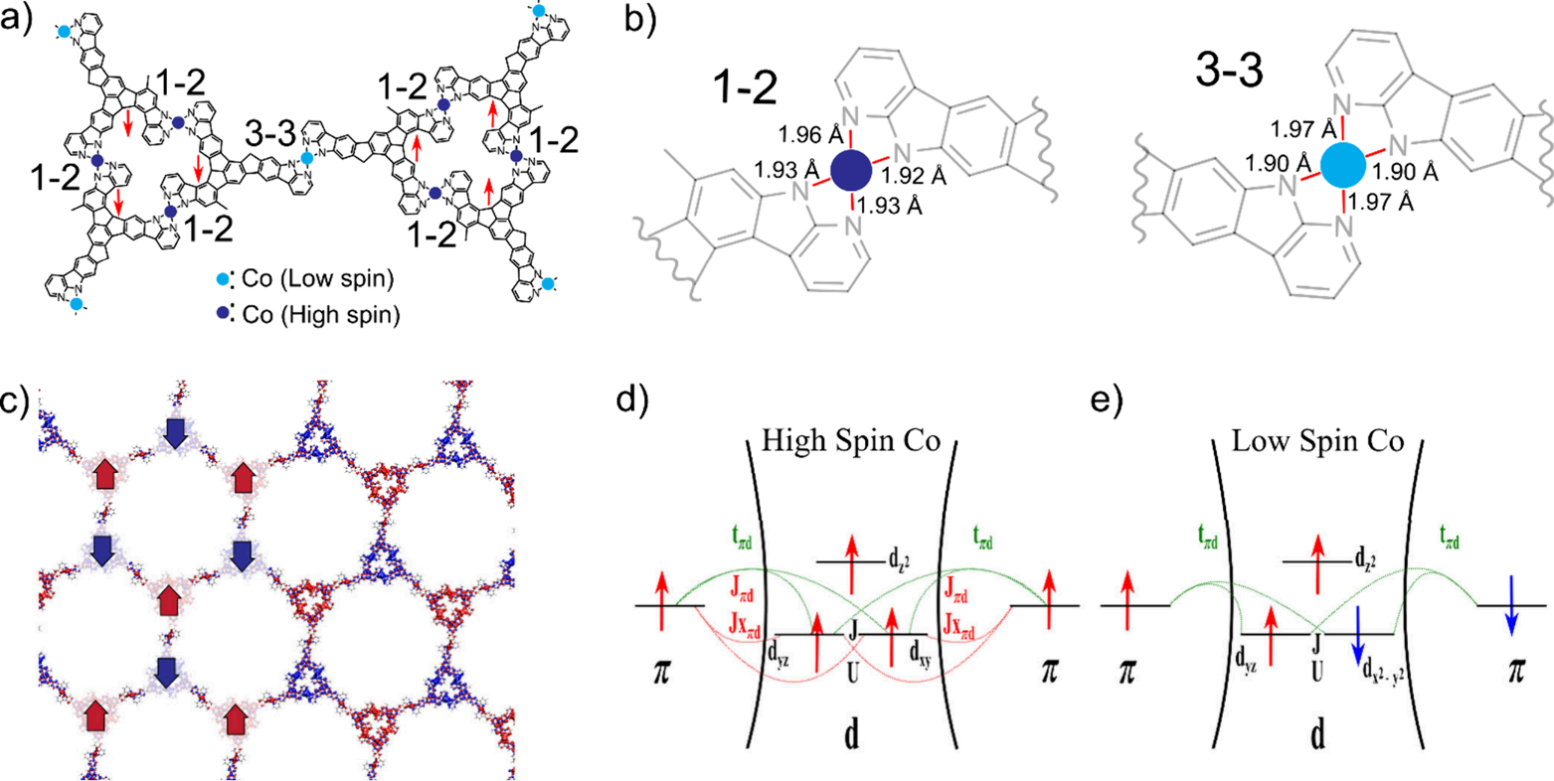
(a) Chemical sketch of two trimers illustrating the spin orientation of the organic radical ligands and showing two types of connections: 1–2 and 3–3 couplings, mediated by low (light blue circles) and high spin (dark blue circles) Co centers, respectively. (b) Bond lengths of the Co–N bond for both 1–2 and 3–3 couplings, obtained from the spin-polarized DFT calculations. (c) Calculated spin density reveals the interplay between ferromagnetic coupling within the trimers and antiferromagnetic coupling between trimers. Red and blue densities indicate spin-up and spin-down contributions, respectively. (d) Schematic representation of coordination of π-radicals through the high spin cobalt providing FM superexchange between the π radicals from the model Hamiltonian. (e) Schematic representation of coordination π-radicals through the low spin cobalt and originating of AFM superexchange between the π-radicals from the model Hamiltonian.

Therefore, we carried out more accurate but computationally expensive multireference CASCI­(11,11) calculations of clusters made of two ligands coordinated by a single Co center in 1–2 and 3–3 dimers (analogous to the experimentally measured ones), using the optimized structure obtained from DFT calculations (see Supplementary Note for details). Our CASCI­(11,11) calculations reveal the presence of 5 unpaired electrons for both dimers based on the occupancy of natural orbitals, which show strong mixing of π and d orbitals; see Figure S20. The ground states of the dimers show strong multireference character with many Slater determinants contributing to the electronic ground state; see Figure S21. Moreover, the CASCI calculations confirm the existence of the different exchange interactions for both dimers. In the case of the dimer 3–3, we obtained an antiferromagnetic coupling between the π-radicals mediated by the cobalt atom in a low spin state with three unpaired electrons, of which one has an opposite spin orientation. In the case of the dimer 1–2, we found a ferromagnetic coupling between the π-radical ligands and the cobalt centers in a high spin state with 3 unpaired electrons per cobalt center. Furthermore, the d^2^ *I*/d*V* ^2^ maps from Figure 3e,h nicely match the simulated natural transition orbitals^46^ shown in Figure 3d,g corresponding to the process of spin excitation from the doublet ground state to the first excited quartet (8 mV) for the dimer 3–3 and the sextet ground state to the first excited quartet (4 mV) for the dimer 1–2, respectively.

Next, we carried out CASCI­(12,12) calculations of trimer 1–2 (see Figure S23), whose results we compared with single reference DFT calculations. According to CASCI calculations, we found a ferromagnetic ground state with 3 Co centers in high-spin states, in good agreement with DFT calculations using PBE0 ^47^ functional with enhanced exact exchange contribution; for details, see discussion in Supplementary Note. This good agreement partially validates the results of the single reference DFT calculations.

According to multireference CASCI calculations of cluster, high-spin Co centers with 3 unpaired d-electrons facilitate the ferromagnetic coupling with π-radicals on neighbor ligands, while low-spin Co centers with 3 unpaired d-electrons impose antiferromagnetic coupling between π-radicals. The origin of these two magnetic interactions cannot be simply explained by the standard superexchange interaction involving the d- and π-orbitals. To obtain more insight into the exchange mechanism between three unpaired d-electrons settled in high/low spin state configuration with the nearest neighbor π-radicals, we analyzed a model Hamiltonian mimicking many-body interaction between localized d-states and extended π-states shown in Figure 4d,e. A detailed description is provided in the Supplementary Note. In summary, both (anti)­ferromagnetic exchange mechanisms for low/high spin Co centers can be described as variants of the double exchange mechanism, with the interplay of kinetic exchange between π and d states and Coulomb exchange between d orbitals. According to the model, the antiferromagnetic interaction between π-states mediated by low-spin Co center (see Figure 4e) can be accomplished by hopping t_πd_ between π and d-orbitals exceeding 0.6 eV. Interestingly, to achieve the ferromagnetic order between π-states mediated by high-spin Co center (see Figure 4d), we have to include besides the hopping t_πd_ also exchange interaction between π–d orbitals.

## Conclusions

We have presented a two-step synthetic process to form atomically precise, large-scale 2D magnetic MOFs. We show that the presence of π-radical ligands mediating the exchange coupling between the metal centers results in a complex electronic configuration with multiple possible spin states and several low-energy excitations. Our fundamental understanding of the π–d interactions between the metal and the ligands allowed us to rationalize the magnetic ground state of the 2D MOF. We experimentally observed the presence of two distinct π–d magnetic interactions, which are rationalized with CASCI calculations. The asymmetric structure of the radical ligand triggers complex ferromagnetic/antiferromagnetic order within the MOF. This work demonstrates the growth of a 2D MOF with different spin arrangements and larger magnetic strengths while preserving a large porosity and high quality, leading to fascinating magnetic functionalities in MOFs and contributing to the development of 2D magnets. We believe that this two-step synthetic process based on a supramolecular organization represents an advance in the large-scale synthesis of 2D MOFs, which can be exploited in different synthetic methodologies and combined with different functionalities, expanding their possibilities.

## Supplementary Material



## Notes

Experimental details, synthesis, characterizations, and computational studies (PDF)
