# Supplementary material for: On-Surface Synthesis of a Large-Scale 2D MOF with Competing π–d Ferromagnetic/Antiferromagnetic Order
Source: J Am Chem Soc. 2025 May 30;147(23):19575–82. doi: 10.1021/jacs.4c17993 (PMC12164333; doi:10.1021/jacs.4c17993)
Supplement: Supplementary file 1 [file ja4c17993_si_001.pdf]

Supporting Information: On-surface synthesis of a large-scale 2D MOF with competing $\pi - \mathrm{d}$ ferromagnetic/antiferromagnetic order

Federico Frezza,[†,‡,#] Manish Kumar,[†,¶,#] Ana Sánchez-Grande,[*,†] Diego Soler-Polo,[†] Manuel Carrera,[§] Oleksandr Stetsovych,[†] Pingo Mutombo,[†,‖] David Curiel,[§] and Pavel Jelinek[*,†,⊥]

†*Institute of Physics, Academy of Sciences of the Czech Republic, Cukrovarnická 10, 162 00, Prague, Czech Republic*

‡*Faculty of Nuclear Sciences and Physical Engineering, Czech Technical University in Prague Břehová 78/7, 11519 Prague 1, Czech Republic*

¶*Department of Condensed Matter Physics, Faculty of Mathematics and Physics, Charles University, Prague 2, CZ 12116, Czech Republic*

§*Department of Organic Chemistry, University of Murcia Campus of Espinardo, 30100 Murcia, Spain*

‖*Département de Raffinage et Pétrochimie, Faculté de Pétrole, Gaz et Énergies Renouvelables, Université de Kinshasa, BP 127, Kinshasa XI, République Démocratique du Congo*

⊥*Regional Centre of Advanced Technologies and Materials, Czech Advanced Technology and Research Institute (CATRIN), Palacký University Olomouc, 779 00 Olomouc, Czech Republic*

#*These authors contributed equally*

E-mail: sanchez@fzu.cz; jelinekp@fzu.cz

Contents

1 Methods **S-3**

Sample preparation S-3

STM/ncAFM measurements S-3

Theoretical Methods S-4

2 Experimental Results **S-5**

Experimental Note: S-10

3 Supplementary note: Theoretical Results **S-15**

Topology of 2D MOF S-15

Analysis of complex $\pi - d$ exchange interaction with model Hamiltonian S-19

References **S-30**

1 Methods

Sample preparation

The synthesis of molecular precursor I is described elsewhere.[S1] The Au(111) substrate (MaTeck GmbH) was cleaned by repeated cycles of Ar+ ion sputtering (E = 1 keV) and subsequent annealing to 740 K for 10 minutes. Molecular precursor I was thermally sublimed (sublimation temperature of 330°C) onto the clean Au(111) surface kept at RT, and subsequently annealed at 325 °C to obtain the radical SOF. Cobalt atoms were deposited employing a EFM 3, Focus GmbH onto the Au(111) surface kept at RT, followed by a subsequent annealing at 300 °C.

STM/ncAFM measurements

The SPM experiments were performed in a ultra-high vacuum system (base pressure below 5×10^{-10} mbar) hosting a commercial low-temperature (1.2 K) STM/nc-AFM microscope (Specs-JT Kolibri sensor: $f \approx 1 MHz$). Unless otherwise noted, All SPM images were taken with a CO-functionalized tip and at ≈ 3K. In nc-AFM images, the frequency shift from the resonance of the sensor (employing a CO-functionalized tip) was recorded in constant-height mode (Nanonis SPM for Createc GmbH). Scanning parameters are specified in each figure caption. Nickelocene (Nc) was deposited from a CF borosilicate glass at room temperature directly onto the sample in the microscope head at <4 K. To pick the Nc, a sample area containing multiple molecules was scanned at a low current and bias set point (<50 pA, 1 mV) until spontaneous functionalization occurred. After the functionalization of the metallic tip with Nc, d^2I/dV^2 spectra recorded on the bare Au(111) revealed two IETS peaks at $f \approx 3.9$ mV.[S2] The Nc-functionalized tip was tested by Δf/z spectroscopy, considering stable tips those exhibiting no hysteretic behavior for forward and backward curves. The STM and nc-AFM images were analyzed using WSxM.[S3]

Theoretical Methods

Density Functional Theory: The geometry optimization of 2D MOF, both in its free-standing form and adsorbed on the Au(111) surface, was performed using Density Functional Theory (DFT) as implemented in the FHI-AIMS code.[S4] Localized atom-centered basis functions with the "light" tier setting, in FHI-AIMS for individual atomic species, were employed. Initially, the equilibrium geometry of the isolated 2D MOF was determined by fully relaxing the unit cell while imposing a planar constraint along the z-axis. The Perdew-Burke-Ernzerhof (PBE)[S5] version of the GGA was employed for the exchange-correlation functional. Owing to the large unit cell dimensions, Brillouin zone integration was restricted to the Γ -point, and calculations were performed within the spin-collinear parameter. The optimized free-standing geometry of 2D MOF was subsequently positioned onto a 22 × 22 supercell representation of a single-layer Au(111) substrate, where the molecular structure was allowed to undergo full relaxation while enforcing planarity of the Au(111) monolayer. The geometry optimization continued until the maximum residual atomic force was below $1 \times 10^{-2} eV/\mathring{A}$. To accurately account for dispersion interactions at the molecule-metal interface, van der Waals corrections were incorporated via the Tkatchenko-Scheffler formalism with Hirshfeld partitioning,[S6] while excluding direct Au-Au interaction. Furthermore, scalar relativistic effects were accounted for in all calculations through the atomic scalar zeroth-order regular approximation (ZORA) method. For the single point calculation, the PBE0 exchange-correlation functional[S7] and single k-point (Γ) were used to sample the Brillouin zone owing to the very large size of the unit cell.

Complete Active Space Configuration Interaction: In addition, we performed the many-body Complete Active Space Configuration Interaction (CASCI) method for selected cluster models to describe more precisely the electronic structure of MOF and to benchmark DFT results. For the CASCI calculations, we have used the natural orbital obtained from the DFT calculation with the PBE functional[S5] as they provide the most compact representation of correlated wavefunctions.[S8] From these DFT natural orbitals, we have constructed

one- and two-body integrals with the quantum chemistry code ORCA.[S9] From these integrals, we have built the many-body Hamiltonian and diagonalized it with our in-house code. The one-particle density matrix (1PDM) was constructed for the ground state to get the natural occupation and orbitals which tell us about the radical character in the many-body context. For both the dimers, we have employed the active space of 11 electrons in 11 orbitals (CASCI(11,11)) which contain three d orbitals of Co atoms hosting the unpaired electrons, and 2 SOMOs of organic radicals and other 6 π orbitals near the Fermi level. To rationalize the experimental IETS spin excitation maps, we calculated the Natural Transition Orbitals (NTOs)[S10] for corresponding excitation from the ground to the excited many-body states.

2 Experimental Results

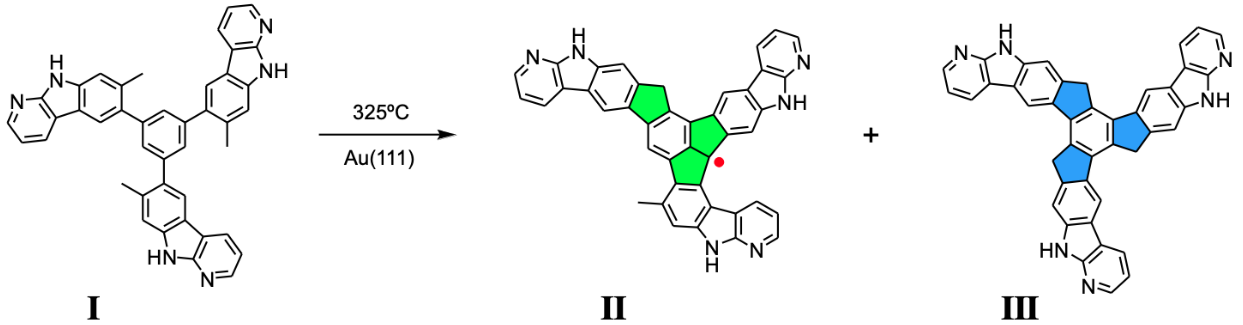

Figure S1: Synthetic route toward the formation of II (organic radical ligand) and III on Au(111).

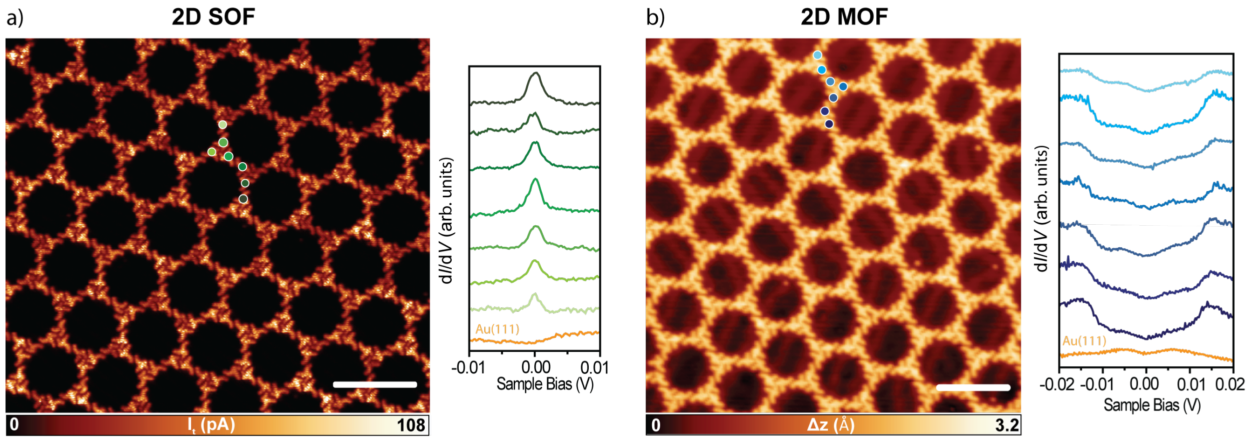

Figure S2: Comparison of the magnetic properties of the 2D SOF and 2D MOF on Au(111). a) Constant-height overview STM image of the 2D SOF on Au(111) and low-energy dI/dV spectra acquired over a series of 7 adjacent molecules confirming the existence of a Kondo resonance peak in all molecules (V_b = 5 mV and scale bar = 8 nm). b) Constant-current STM image of the cobalt coordinated 2D MOF on Au(111) and low-energy dI/dV spectra acquired over a series of 7 adjacent ligands confirming spin interactions (V_b = 100 mV, I_t = 20 pA and scale bar = 7.5 nm).

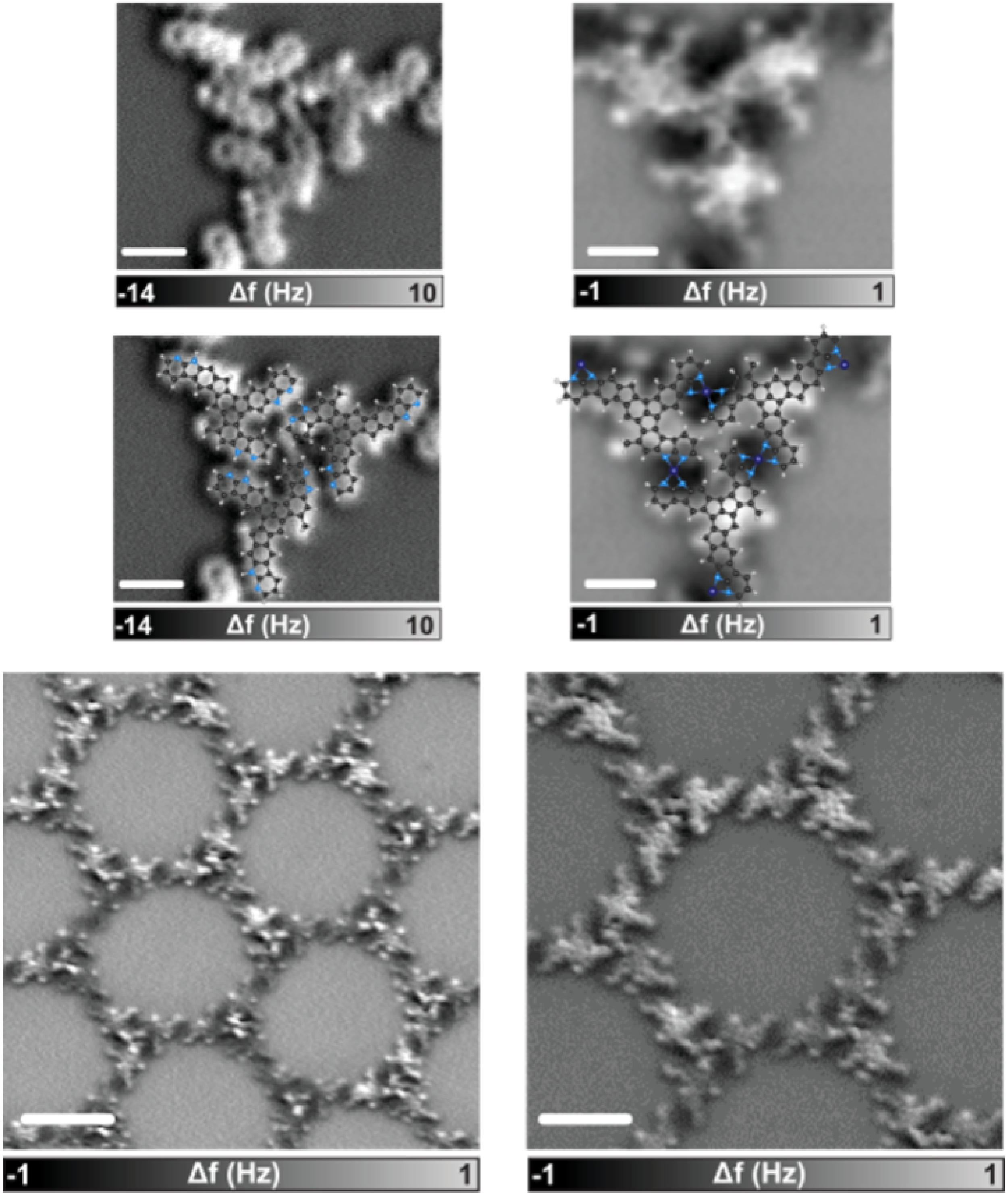

Figure S3: Top panel: NcAFM images from Figure 1f,g with a model superimposed for better visualization. Bottom panel: nc-AFM images of the 2D MOF on Au(111), confirming the Co-coordination. (Right panel: $V_b = 1$ mV, scale bar = 3.2 nm. Left panel: $V_b = 1$ mV, scale bar = 2.2 nm).

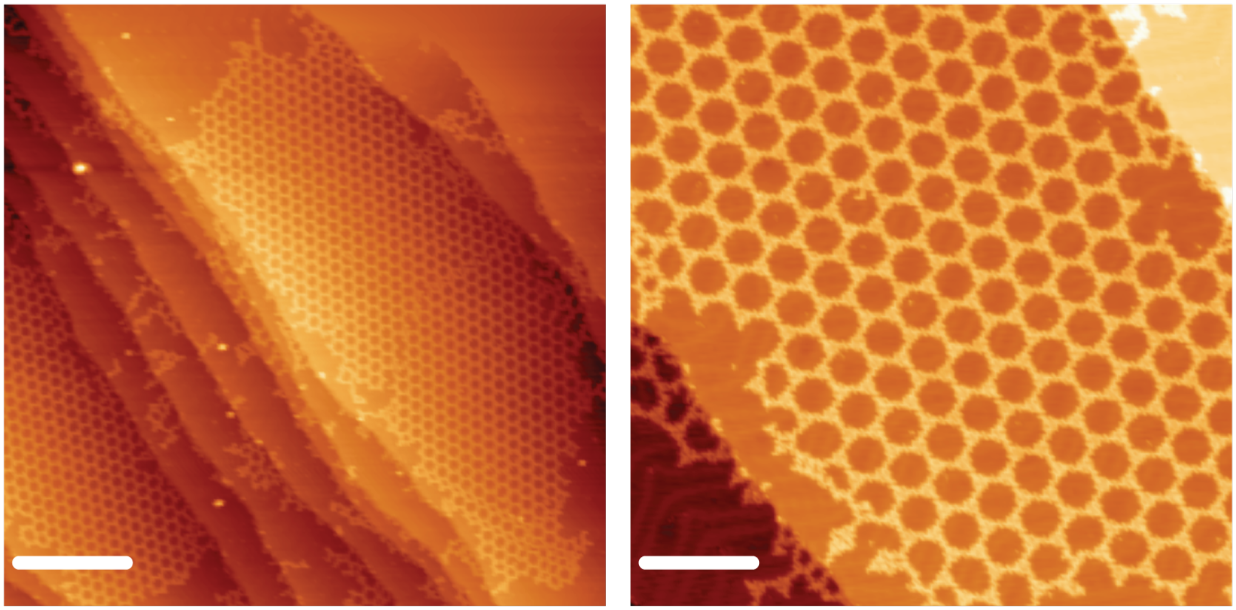

Figure S4: STM overview images showing large scale 2D MOFs on Au(111). Left panel: scale bar = 50 nm, V_b = 75 mV and I_t = 16 pA. Right panel: scale bar = 16 nm, V_b = 80 mV and I_t = 20 pA.

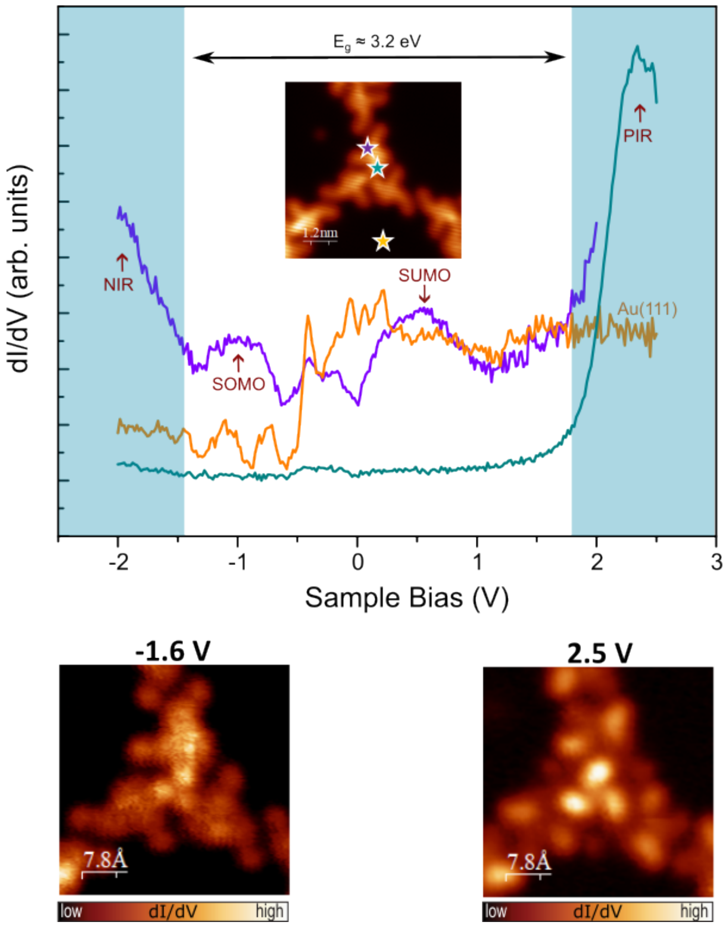

Figure S5: Differential conductance dI/dV spectra acquired over the ligands of the 2D MOF and on the Au(111) as reference. The spectra positions are depicted in the inset STM image. Bottom panel show the experimental dI/dV maps acquired at -1.6 eV and 2.5 eV.

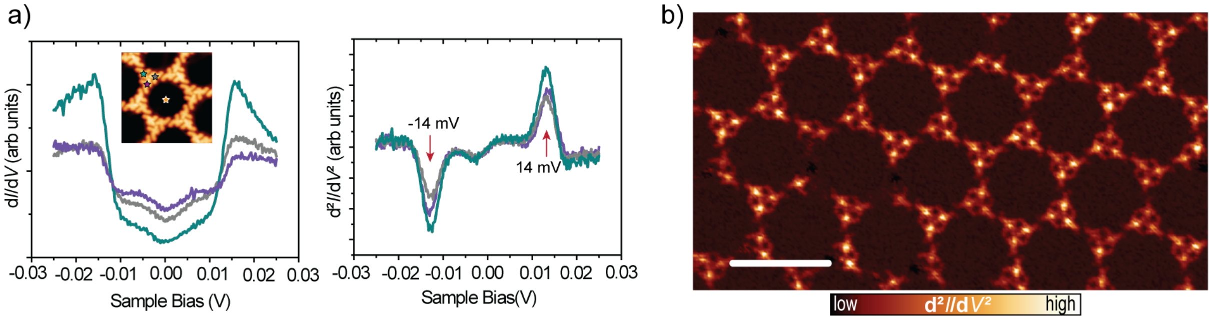

Figure S6: a) Low-energy dI/dV and d^2I/dV^2 spectra acquired over the organic ligand forming one trimer (positions depicted in inset STM image). b) Experimental constant-height d^2I/dV^2 spin excitation map (V_b = 14 mV and scale bar = 7.0 nm).

Experimental Note:

The experimental evidence of the ligand-directed growth of the 2D MOF ("using" the radical SOF as a template) is based on different aspects:

- The sample preparation consists of i) the formation of the 2D SOF, ii) Co atoms deposition, and iii) annealing at 300 °C to induce metal coordination. We can follow the step-wise process to get insights into the influence of the Co deposition on the supramolecular framework. Figure S7 shows a comparison of the same sample after forming the 2D SOF(left panel) and after Co deposition (right panel). Interestingly, after Co deposition the supramolecular organization is preserved intact. Thus, we can conclude that the cobalt impact energy does not affect the 2D SOF.

- Previous work about the study of the thermal stability of a 2D SOF containing benzodi-7-azaindole,[S11] where each molecule presents four hydrogen bonds with the adjacent molecules, demonstrates thermal stability up to 300°C. In the case of our 2D SOF, each molecule sets six reciprocal hydrogen bonds through the three azaindole units, giving extra thermal stability compatible with the temperatures needed to induce thermal coordination.

- The possibility of forming only a partially metalled sample with a low amount of Co centers randomly distributed over the molecular network (see Figure 3 in the manuscript) rules out the scenario involving the dissolution of the SOF phase and subsequent formation of the 2D MOF phase proposed by the referee.

- If we assume that the supramolecular organization dissolves, we expect to see different coordination possibilities, as shown in Figure S8. However, we do not observe these connections in our experiments.

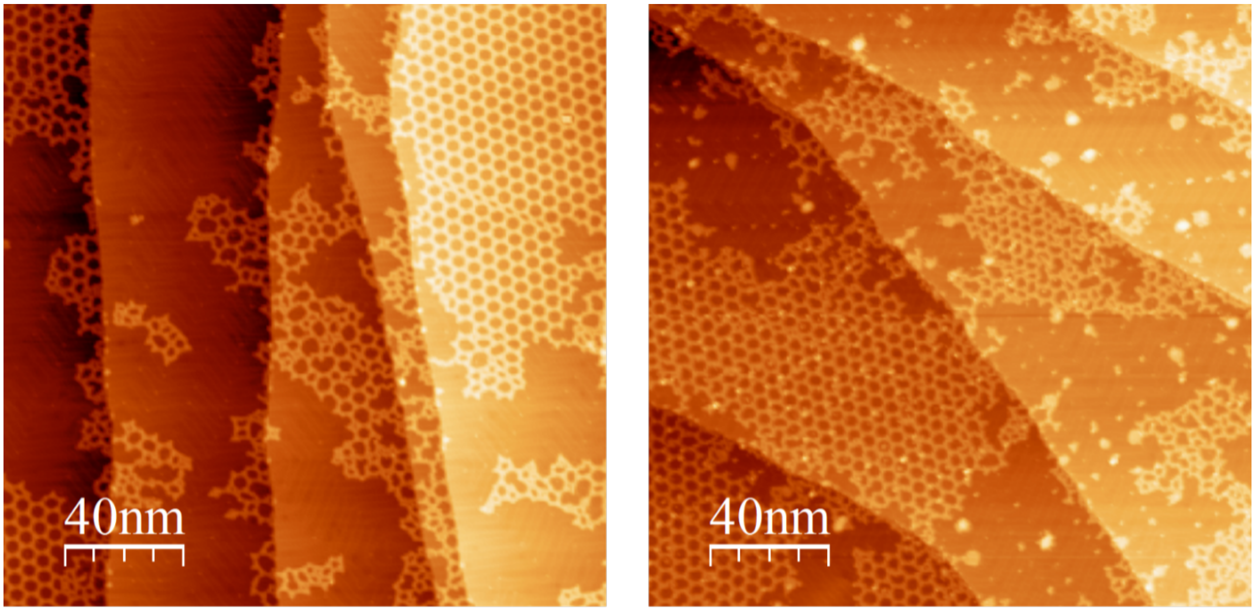

Figure S7: Left panel: 2D SOF on Au(111). Right panel: same sample after Co deposition.

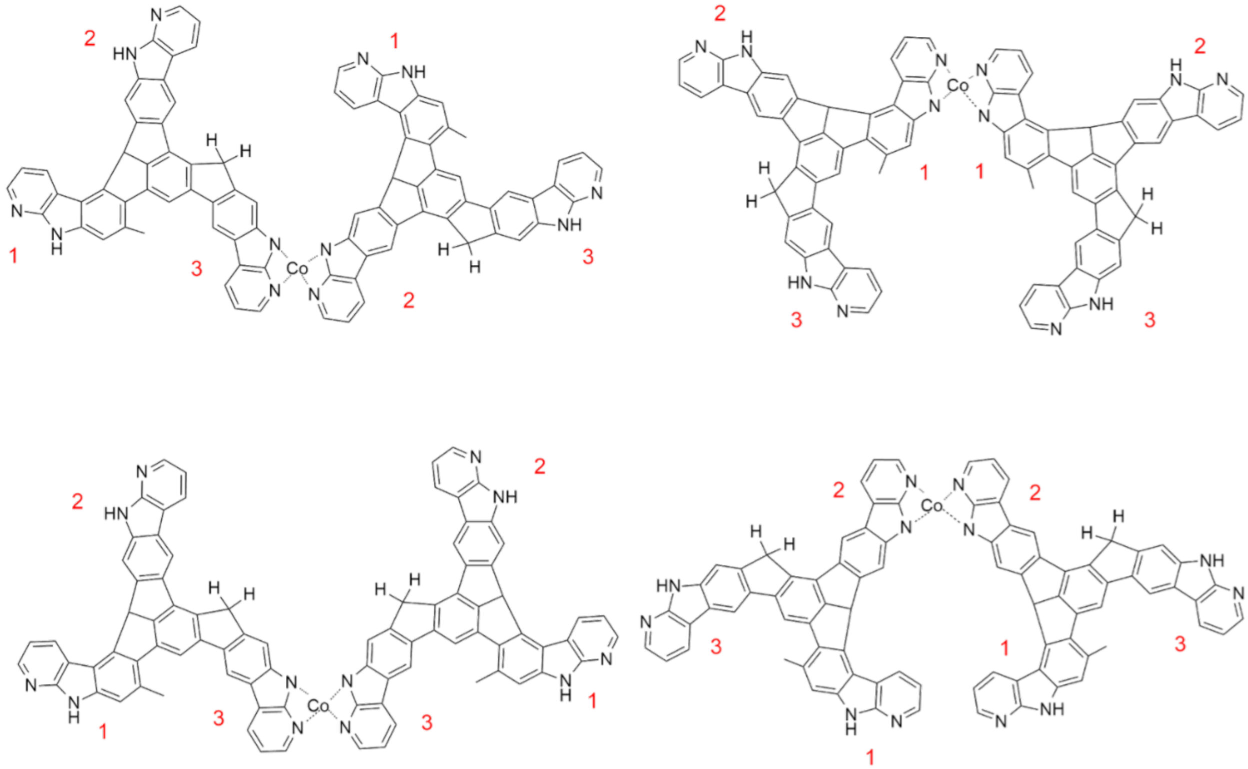

Figure S8: Chemical sketches of different hypothetical connections after Co coordination.

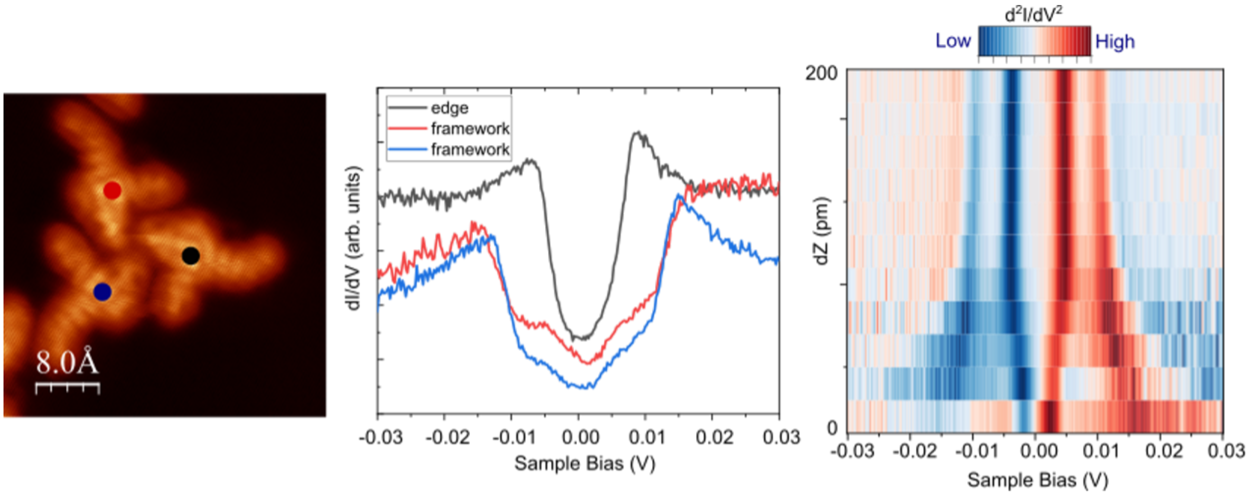

Figure S9: Comparison of the magnetic properties of the ligands within the frameworks (blue and red spectra), characterized by a V-shape feature at 14 mV, with respect to a molecule at the edge (black spectrum), presenting U-shaped dip at $\approx$ 6 mV. The right panel corresponds to the d^2I/dV^2 spectra plotted in a color map as a function of the tip-sample distance acquired over the ligand at the edge employing a nickelocene-functionalized tip.

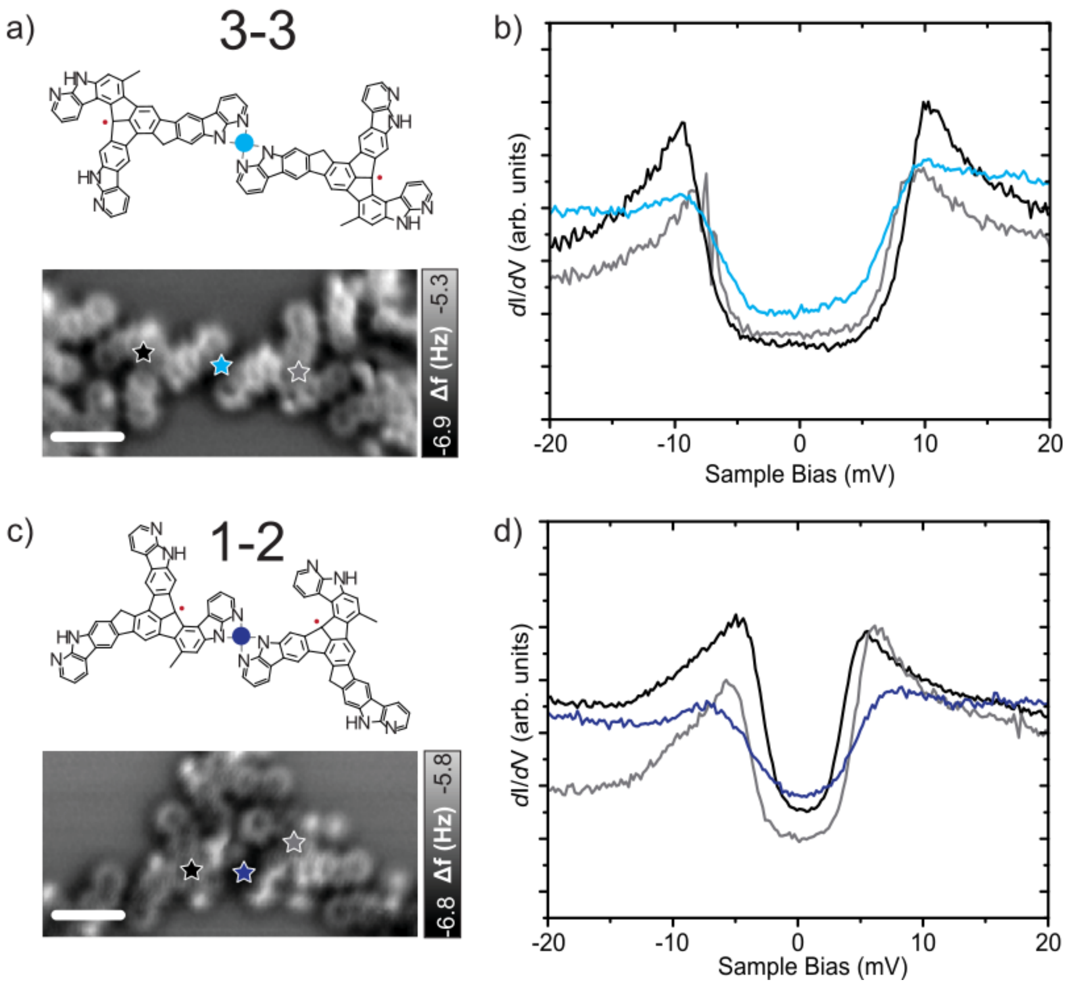

Figure S10: Differential conductance spectra acquired on the ligand-Co-ligand positions of the model dimers with 3-3 and 1-2 couplings. a) Chemical sketch and nc-AFM of the 3-3 coupling. b) dI/dV spectra acquired on both ligands and on the Co atom (positions marked in the nc-AFM image). c) Chemical sketch and nc-AFM of the 1-2 coupling. d) dI/dV spectra acquired on both ligands and on the Co atom (positions marked in the nc-AFM image).

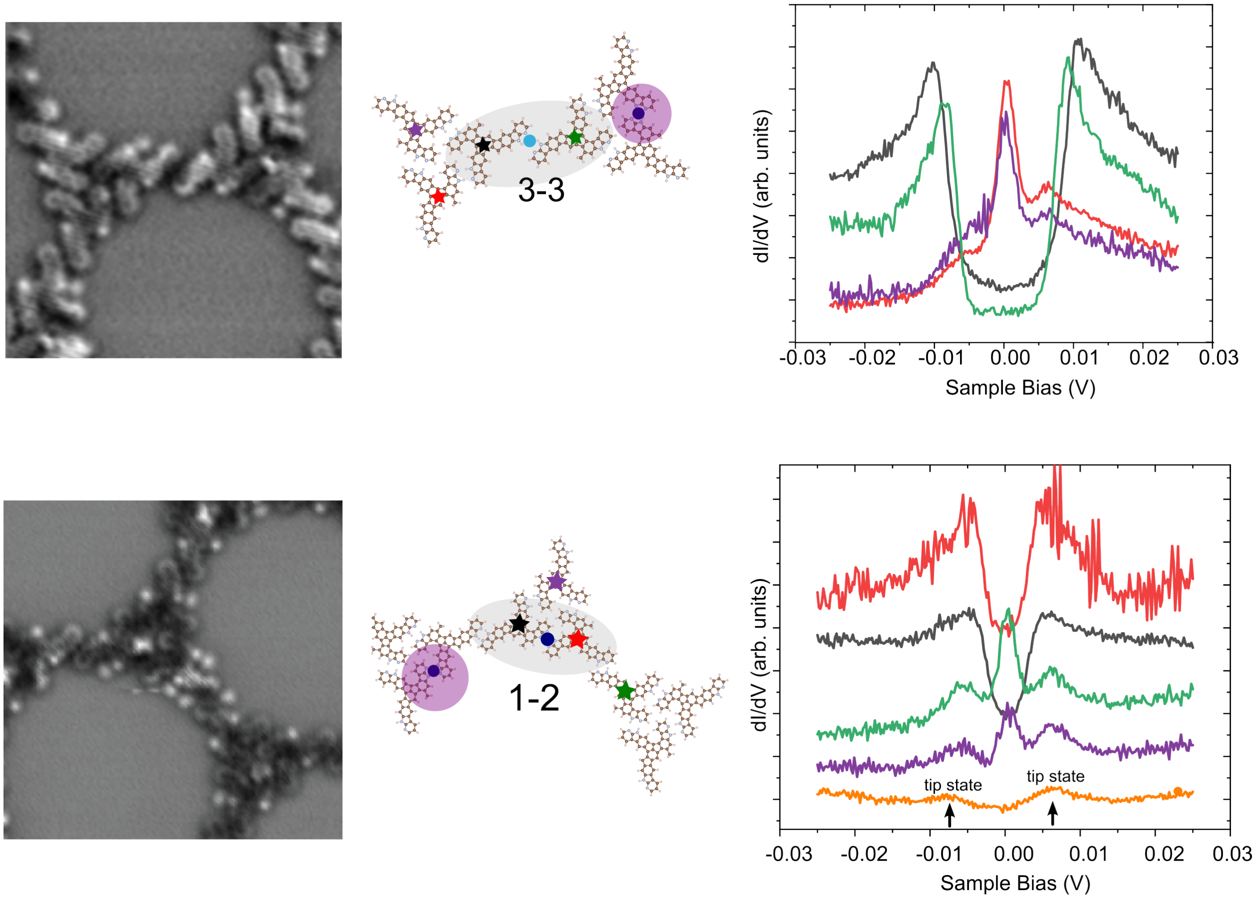

Figure S11: The top panel shows the nc-AFM image, model and STS measurements (spectra positions depicted in the model as stars following the color code) of 3-3 dimers (light blue ball correspond sts low spin Co), showing the electronic properties of the adjacent molecules. Bottom panel shows the nc-AFM image, model and STS measurements (spectra positions depicted in the model as stars following the color code) of 1-2 dimers (dark blue balls correspond to high spin Co), showing the electronic properties of the adjacent molecules. As shown in the figure, even if these adjacent ligands are cobalt coordinated to an additional ligand (violet circles in the scheme), this will not affect the signal of the dimer under study, since the hydrogen-bonding interactions keep the dimers "isolated", as evidenced by the presence of the Kondo peaks for the molecules adjacent to the 1-2 or 3-3 dimers that are not coordinated to any cobalt atoms.

3 Supplementary note: Theoretical Results

Topology of 2D MOF

Figure S12c displays SOMO orbital of the ligand revealing its different distribution across three 7-azaindole units, labeled as 1, 2, and 3. From the high-resolution nc-AFM images of 2D MOF, shown in Figure S3 and Figure 2a of the main text, we can deduce the chemical structure of 2D MOF. Consequently, we can identify two different types of Co atoms according to their coordination with the organic radical ligand, see Figure S12a. One type of Co (labeled by dark blue) is located inside the trimer unit and coordinated with the organic ligand through two 7-azaindole units, labeled 1 and 2. Thus, we label it as 1-2 coupling. The second type of Co (labeled by light blue) is located between the two trimer units and coordinated with the organic ligand through two identical 7-azaindole units, labeled 3, forming a 3-3 coupling. A chemical sketch of this coordination in the unit cell is shown in Figure S12b. Within the unit cell, only these two types of couplings 1-2 and 3-3 exist. We should stress that all 1-2 couplings are equivalent, as they occur in the same manner and are related by a 120° rotation.

DFT results:

We employed total energy spin polarized DFT calculations to obtain fully optimized atomic structure of free standing 2D-MOF. Figure S12d displays the optimized Co-N bond lengths for the 1-2 and 3-3 couplings, revealing the presence of an asymmetric ligand coordination. From the spin-unrestricted DFT calculations for the periodic 2D MOF, it is found that there are ferromagnetic couplings between the π-radicals and d electrons of Co in a high-spin state forming the 1-2 coupling. while there is anti-ferromagnetic coupling between the π-radical coordinating with low-spin Co forming the 3-3 coupling. Figure S13 represents the spin density obtained from DFT revealing strong delocalization of the spin density over both the ligand and Co centers with alternating spin orientation on the adjacent trimers. Thus, spin polarized DFT calculations reveal complex long-range order of the 2D MOF with alternating

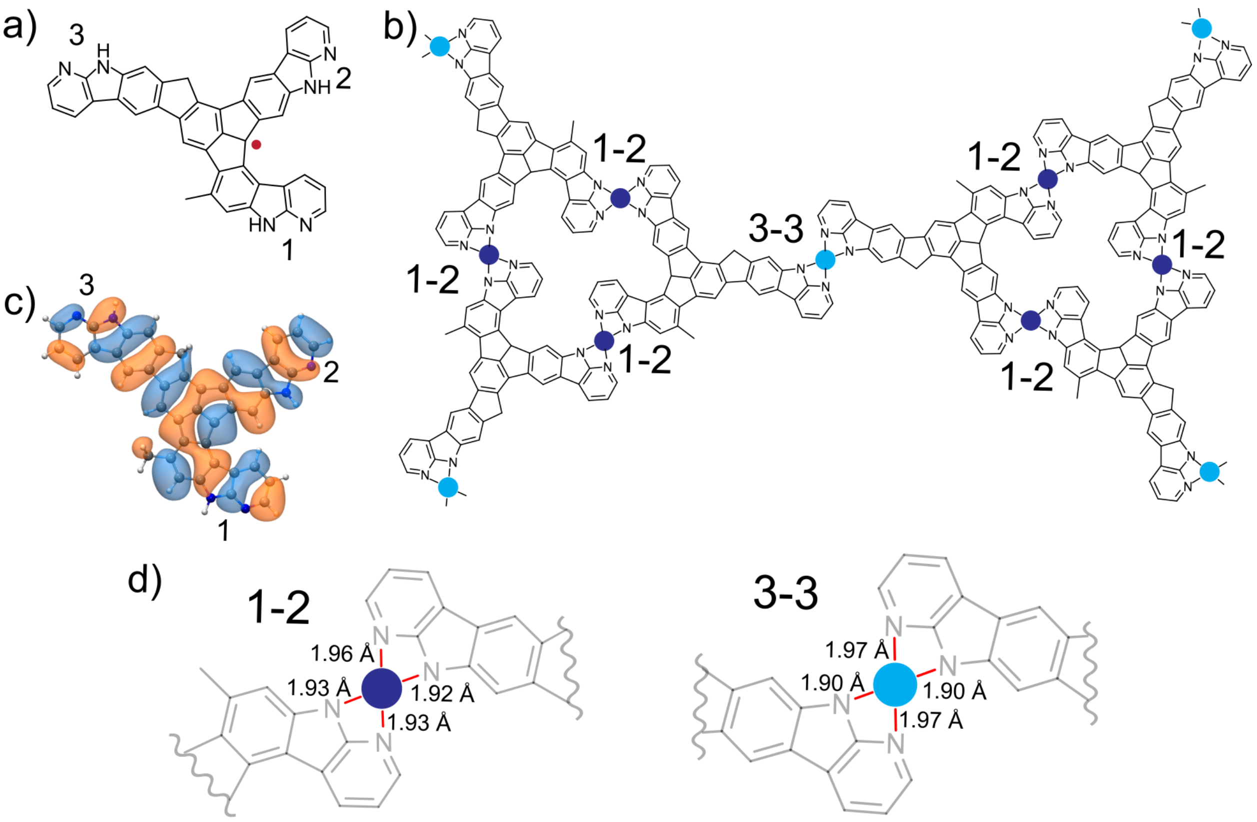

Figure S12: (a) Chemical sketch of the organic radical ligand. (b) Chemical representation of the unit cell, illustrating the coordination of organic ligands with Co atoms and the two possible coordination scenarios, marked with two different colors. (c) Simulated SOMO orbital of the organic ligand. (d) Bond lengths of the Co–N interactions for both 1-2 and 3-3 couplings, obtained from the DFT-PBE optimized geometry.

FM and AFM order.

Moreover, to understand the effect of the underlying Au(111) surface, we also carried out the DFT calculation including one layer of Au(111) surface. Figure S18 shows the side view of the optimized structure revealing only negligible vertical relaxation of 2D MOF. Individual Co atoms are located ≈ 3.12Å above the Au(111) surface indicating a physisorption regime.

CASCI results:

The question is whether the single reference DFT method can accurately describe the character of the electronic structure of the MOF, due to the presence of unpaired electrons on both the organic ligand and Co atoms. For this purpose, we performed multireference CASCI

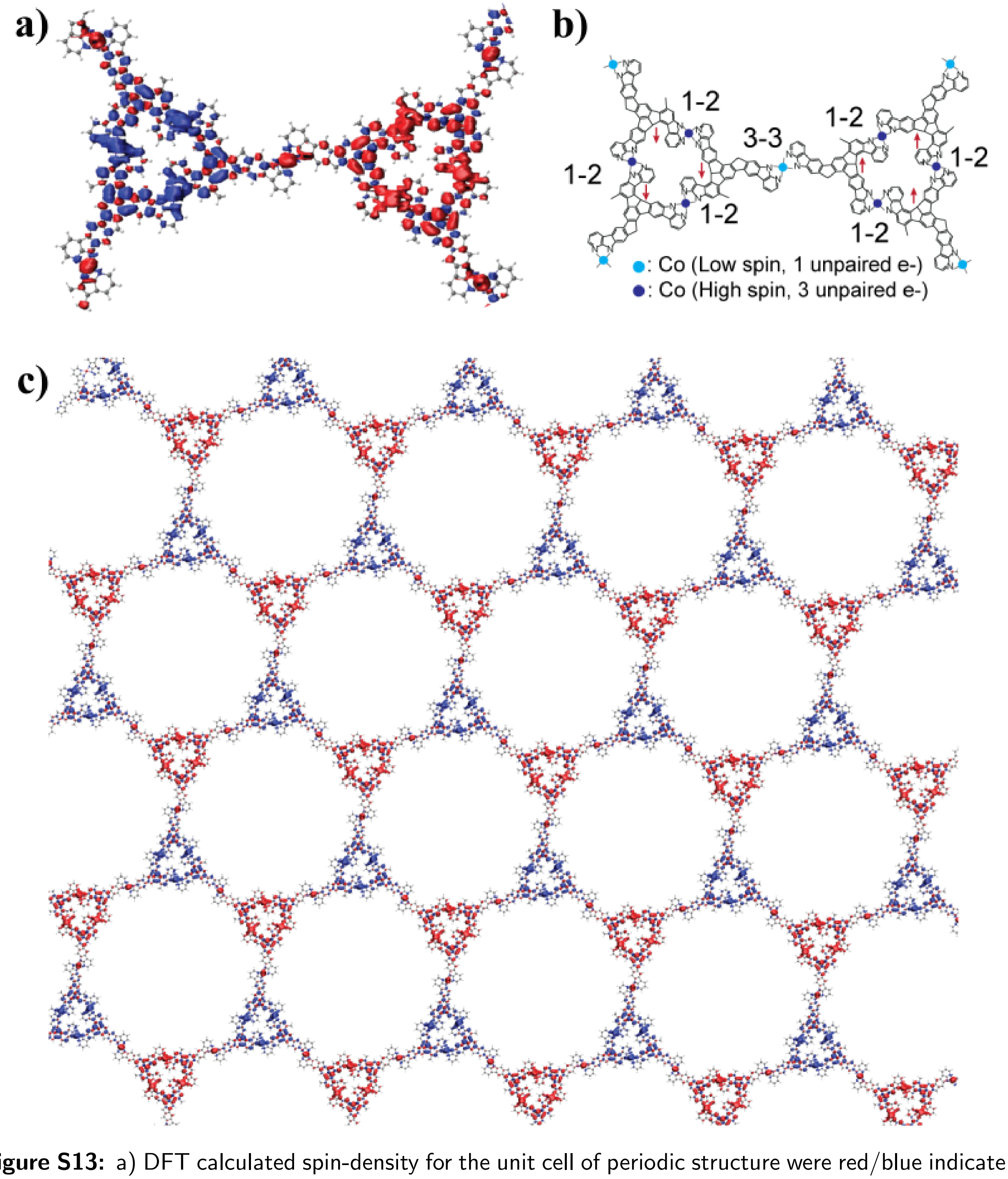

Figure S13: a) DFT calculated spin-density for the unit cell of periodic structure were red/blue indicate the density of spin up/down respectively ; b) chemical sketch of unit cell of periodic MOF with the schematic spin orientations; c) Spin-density of the super-cell of periodic MOF with red/blue color denotes the density of spin up/down respectively.

calculations. However, CASCI method allows calculations only on non-periodic structures and is computationally very demanding. Therefore, we performed CASCI calculations on

selected model clusters, the geometry of which was derived from DFT calculations. Namely, we considered two dimers, each of them representing the characteristic coupling motif 1-2 and 3-3 represented in the 2D MOF, as shown in Figure S14. This enables us to get more insight into the local electronic and magnetic structure of the given coupling motif.

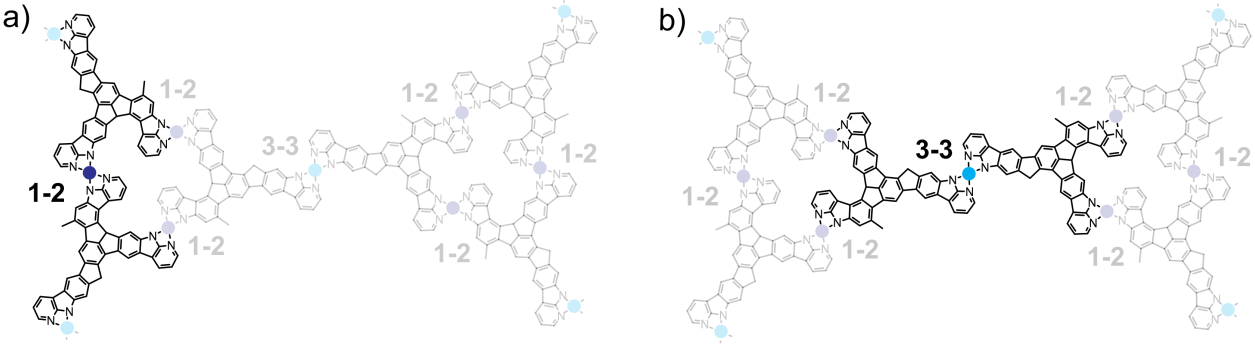

Figure S14: Two dimers selected to investigate the different coupling using the many-body CASCI calculations: (a) 1-2 coupling and (b) 3-3 coupling, as observed in the 2D MOF structure.

Dimers 1-2 and 3-3:

We performed CASCI(11,11) calculation for two dimers labeled 1-2 and 3-3 in the bases of DFT natural orbitals shown in Figures S19a and S19b respectively. The occupancy of calculated natural orbitals obtained from the CASCI calculation, shown in Figures S20a and S20b reveals the presence of five singly occupied natural orbitals (SONO) with occupancy ≈ 1.0 in both cases. We also observe significant contributions of both d and π orbitals to SONOs, pointing out the presence of strong $\pi - d$ coupling. In the case of the 1-2 dimer, we identified the dominant presence of d_{YZ}, d_{XY} and d_{Z^2} orbitals of Co atom in SONO, while in the case of the 3-3 dimer, we found three unpaired electrons presented in d_{YZ}, d_{Z^2}, and $d_{X^2-Y^2}$ orbitals of Co atom.

The ground states of both dimers show strong multireference character with similar contributions of many Slater determinants as shown in Figure S21. The dimer 1-2 has a high-spin sextet ground state followed by two quartet states and a doublet state within ≈ 10 mV above the energy of the ground state (see Table S1(left)). The dimer 3-3 has a low-spin doublet ground state followed by a quartet excited state as shown in Table S1(right).

In addition, we simulated dI/dV maps using NTOs corresponding to the transition from the ground state to the first excited state for both dimers 1-2 and 3-3, see Figure S22. Simulated dI/dV maps reveal the dominant contrast on the ligand part of the dimer, which nicely matches the experimental observation.

Trimer 1-2:

In next, we considered trimer 1-2 made of three organic ligands and three Co centers connected via the 1-2 coupling, which represents the core pattern of 2D MOF as shown in Figure S23. We carried out a CASCI(12,12) calculation to gain a better understanding of the interactions throughout the trimer unit which includes three d-orbitals for each Co center (each containing an unpaired electron) and three SOMOs from each organic ligand, as shown in Figure S23. The CASCI calculation reveals that the ground state of the trimer exhibits high-spin ferromagnetic coupling between all twelve unpaired electrons. Table S2 presents a range of states with small energy differences, representing various spin states from the CASCI calculations for the trimer 1-2.

Unfortunately, expanding the size of the cluster model beyond the trimer makes the CASCI calculations computationally intractable. Therefore, we employed the trimer model as a benchmark for single-reference DFT results. Table S3 compares total energies obtained from DFT-PBE0 calculations for different spin configurations of the trimer 1-2 using different portions of Hartree-Fock exchange. We observe that with increasing Hartree-Fock exchange, the trimer unit exhibits overall ferromagnetic coupling. Thus, the DFT-PBE0 results with enhanced Hartree-Fock exchange align well with the CASCI results.

Analysis of complex $\pi - d$ exchange interaction with model Hamiltonian

The DFT and CASCI calculations provide valuable information about the electronic and magnetic structure of 2D MOF. Nevertheless, due to their complexity, they do not reveal the detailed mechanism of the complex exchange interactions between delocalized unpaired

π-electrons of ligand and strongly localized d electrons of Co centers in the 2D MOF. The presence of several delocalized π and localized d unpaired electrons renders a complicated scenario including both Coulomb and kinetic-exchange interactions between them.

To understand this complex $\pi - d$ exchange interaction we employ a model Hamiltonian H, which allows us to describe the electronic interactions and hopping processes between the d-electrons of Co center and π-electrons of organic radicals. The model Hamiltonian H is schematically sketched in Figure S15 and it is given by:

$$H = H_d + H_\pi + H_{\pi-d}. \tag{1}$$

where

$$H_\pi = \varepsilon_\pi \sum_\sigma (n_{1,\sigma} + n_{2,\sigma}), \tag{2}$$

is the Hamiltonian modeling the two ligand sites, each of them hosting one π-radical. Here, $n_{i,\sigma} = c_i^\dagger c_i$, where $c_i^\dagger (c_i)$ creates (annihilates) an electron at site i.

H_d is the Kanamori Hamiltonian,[S12] which captures intra-orbital and inter-orbital Coulomb interactions, Hund's coupling, spin-flip, and pair-hopping terms, given by:

$$\begin{aligned} H_d = &\sum_{m\sigma} \varepsilon_m n_{m\sigma} + U \sum_m n_{m\uparrow} n_{m\downarrow} + (U' - J) \sum_{m \neq m', \sigma} n_{m\sigma} n_{m'\sigma'} \\ &- J \sum_{m \neq m'} d_{m\uparrow}^\dagger d_{m'\downarrow}^\dagger d_{m\downarrow} d_{m'\uparrow} \\ &- J' \sum_{m \neq m'} d_{m\uparrow}^\dagger d_{m\downarrow}^\dagger d_{m'\downarrow} d_{m'\uparrow}, \end{aligned} \tag{3}$$

where:

- U is the intra-orbital Coulomb interaction,
- U' is the inter-orbital Coulomb interaction,
- J is the Hund's coupling, favoring parallel spin alignment,

- J' is the pair-hopping interaction, which enables electron pairs to move between orbitals,
- $d^\dagger_{m\sigma}$ ($d_{m\sigma}$) creates (annihilates) an electron with spin σ in orbital m of the d-shell,
- $n_{m\sigma} = d^\dagger_{m\sigma} d_{m\sigma}$ is the number operator,
- $U' = U - 2J$ and $J' = J$ due to rotational invariance.

The Kanamori Hamiltonian H_d captures the essential multi-orbital correlations that play a crucial role in transition-metal systems.

The $H_{\pi-d}$ interaction is described by:

$$H_{\pi-d} = \sum_{\langle i,j \rangle,\sigma} \left(t_{\pi d} d^\dagger_{i,\sigma} c_{j,\sigma} + \text{h.c.} \right), \quad (4)$$

where $t_{\pi d}$ is the hopping amplitude between the π- and d- orbitals which describes electron transfer between these π and d orbitals. This charge fluctuation between π orbitals and localized d orbitals plays an important role in determining the ground state electronic structure and magnetic order.

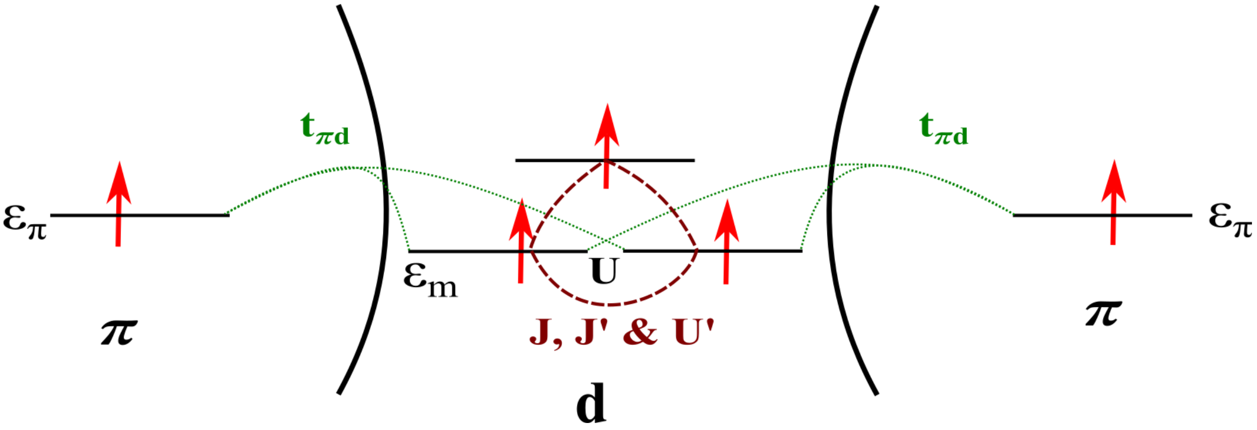

Figure S15: Schematic representation of the Hamiltonian shown in Eq. 2, Eq. 3 and Eq. 4.

The full Hamiltonian $H = H_\text{d} + H_\pi + H_{\pi-d}$ accounts for both the strong electronic correlations in the localized d-orbitals and the hybridization between d- and π-orbitals via

hopping. Thus, this model serves as a foundation for exploring the interplay between orbital-dependent correlations and the charge fluctuation between π and d orbitals. There are several parameters defining the model Hamiltonian.

The Kanamori Hamiltonian of d-orbitals (eq. 3) is constructed directly from ab-initio CASCI calculations of the dimer models considering three unpaired d-orbitals in the active space, as shown in Figure S15. For the hopping $t_{\pi d}$ between d-orbitals and two π-orbitals, we consider it as a variable that determines the magnetic ground state.

We now analyze the magnetic ground state of the model Hamiltonian by considering two cases: low-spin and high-spin Co centers with three singly occupied d-orbitals mediating interactions between two singly occupied π-orbitals, as shown schematically in Figure S15. Our goal is to explore the regions where ferromagnetic (FM) and antiferromagnetic (AFM) interactions emerge between the π-radicals and to understand the role of $\pi - d$ coupling in detail. In both cases, different d-orbitals are involved, leading to the formation of either high-spin or low-spin Co atoms, as discussed in the following part.

Low-spin Co: First, we will discuss the magnetic ground state of the model Hamiltonian for the low-spin Co state. From the CASCI results, there are three unpaired electrons in the d_{YZ}, d_{Z^2}, and $d_{X^2-Y^2}$ orbitals of the Co atom. The Kanamori Hamiltonian in the d-orbitals is constructed by taking the integral of d-orbitals used for the CASCI Hamiltonian, which favors the low-spin of the Co atom. To include the electron hopping between the π-electrons and d-orbitals $t_{\pi-d}$, we have to consider only two orbitals d_{YZ} and $d_{X^2-Y^2}$, as shown in Figure S16a because there is zero overlap between d_{Z^2} and π orbitals due to their symmetry.

Now, we analyze the magnetic ground state of the model Hamiltonian in the parametric space of the hopping $t_{\pi-d_{yz}}$ and $t_{\pi-d_{X^2-y^2}}$. Figure S16b represents a 2D plot of the spin correlations between two π orbitals in the ground state solution as a function of $t_{\pi-d_{yz}}$ on the x-axis and $t_{\pi-d_{X^2-y^2}}$ on the y-axis. Red/blue shows the FM/AFM coupling between the two π electrons. In the region of very small hopping, the two π-radicals favor the FM super-exchange. Increasing the $t_{\pi d}$ hopping, the two π-radicals start to have AFM super-

exchange (blue region in Figure S16b. The AFM configuration can be understood by the strong $\pi - d$ interaction in the system as a white star in spin-correlation maps corresponding to the integral value of $t_{\pi-d}$ obtained to build the full CASCI Hamiltonian.

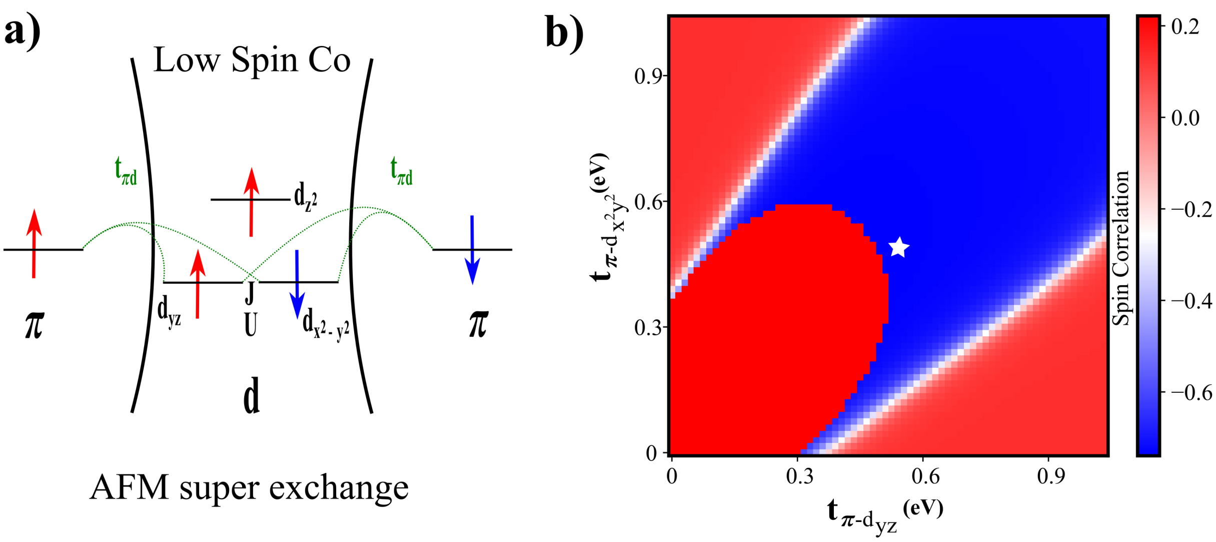

Figure S16: a) Schematic representation of the low spin cobalt bonded to the π radicals. b) 2D color map of spin correlation between the π radicals with $t_{\pi-d_{yz}}$ on x-axis and $t_{\pi-d_{X^2-y^2}}$ on y-axis. Red/blue represent the FM/AFM spin-correlation between the π-radicals and white star is corresponds to the integrals calculated when constructing CASCI Hamiltonian.

High-spin Co: For the case of the dimer 1-2, CASCI calculation shows that there are three unpaired electrons in the d_{YZ}, d_{XY}, and d_{Z^2} orbitals and two unpaired electrons in the π-orbitals. We take the integrals for Kanamori Hamiltonian of d-orbitals of Co atom from the CASCI calculation of the dimer 1-2. This Kanamori Hamiltonian of d-orbitals favors the high-spin of the Co atom. The presence of $d_{X^2-Y^2}$-orbitals in this case differs from the case of the dimer 3-3, where the orbital is d_{XY}.

First, we analyze a model Hamiltonian with the hopping $t_{\pi d}$ between the π system and d-orbitals. In this case, we found that the ground state always has FM superexchange between the π-radicals and AFM coupling between π and d-electrons independently of the magnitude of $t_{\pi d}$ hopping. This simple model is thus able to explain the FM order between the two π radicals in dimer 1-2 mediated by the high-spin Co atom, but it cannot account for the overall FM order of all unpaired electrons predicated by CASCI calculations for the dimer

1-2.

To account for the configuration of the highest-spin state (i.e. FM alignment of both d and π states), we have to expand the interaction $H_{\pi-d}$ to include many-body terms:

$$H_{\pi-d} = \sum_{\langle i,j\rangle,\sigma} t_{\pi d} d^{\dagger}_{i,\sigma} c_{j,\sigma} + \sum_{\langle i,j\rangle,\sigma,\sigma'} J_{\pi d} d^{\dagger}_{i,\sigma} c^{\dagger}_{j,\sigma'} c_{j,\sigma'} d_{i,\sigma} \tag{5}$$

$$+ \sum_{\langle i,j\rangle,\sigma} J_{x,\pi d} d^{\dagger}_{i,\sigma} c^{\dagger}_{j,\sigma} c_{j,\sigma} d_{i,\sigma} + \sum_{\langle i,j\rangle\sigma} J_{x,\pi d} d^{\dagger}_{i,\sigma} c^{\dagger}_{j,\overline{\sigma}} d_{i,\overline{\sigma}} c_{j,\sigma} + \text{h.c.}. \tag{6}$$

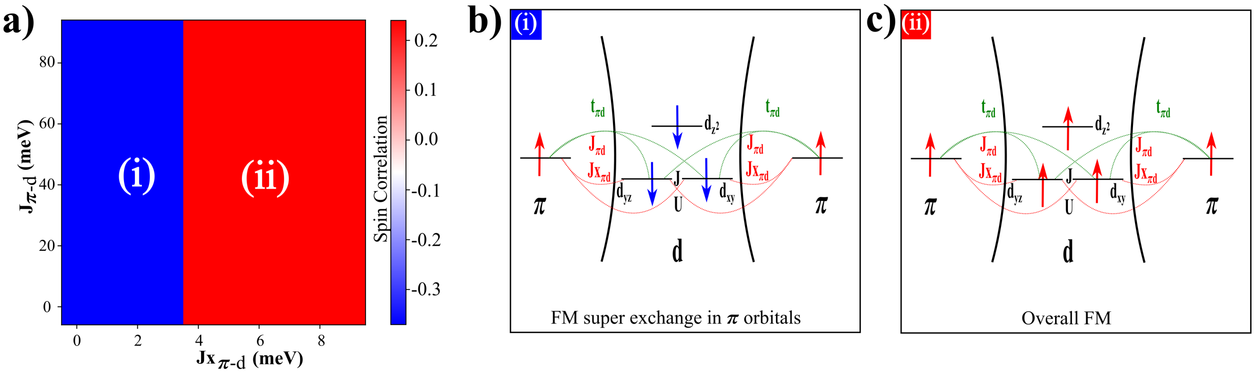

Figure S17: a) 2D color map of spin correlations between the π radical and d-electrons with $Jx_{\pi-d}$ on x-axis and $J_{\pi-d}$ on y-axis which shows the onset of ferromagnetic order between π and d electrons. Red/blue shows the FM/AFM coupling between the π and d system; b) Schematic representation of blue region of a) where there is anti-ferromagnetic order between π and d electrons; c) Schematic representation of red region of a) where there is ferromagnetic order between π and d electrons.

If the parameter $J_{x,\pi d}$ is large enough, it will favor the ferromagnetic coupling between the π and d orbitals independently of parameter $J_{\pi d}$ as shown in Figure S17. In Figure S17 $Jx_{\pi-d}$ is plotted on the x-axis and $J_{\pi-d}$ on the y-axis. Red/blue shows the FM/AFM coupling between the π and d system. Qualitatively, we see a step-wise behavior going from AFM to FM order, in which the ground state of the model Hamiltonian goes from the one described in Figure S17b to Figure S17c. In this way, we see that the explanation of the full ferromagnetic order is mathematically akin to the emergence of Hund's rule in transition metals. Furthermore, the values of $J_{\pi d}$ and $J_{x,\pi d}$ correspond to the Coulomb integrals that we calculate when constructing the CAS Hamiltonian. Such computation yields a $J_{x,\pi d} \approx 10$ mV, which falls in the FM order.

This analysis reveals that the low-spin and high-spin states of the Co atom arise from the involvement of different d-orbitals in the two types of dimers, leading to distinct exchange interactions. The spin state of the Co center—whether low-spin or high-spin plays a crucial role in mediating the exchange interactions between π-radicals. In the case of a low-spin Co atom, the system favors FM superexchange between the π-radicals due to strong πd coupling. On the other hand, when the π system is mediated by a high-spin Co atom, the overall ferromagnetism requires the inclusion of many-body interactions in $H_{\pi-d}$, which, like in the low-spin case, also originates from strong πd coupling.

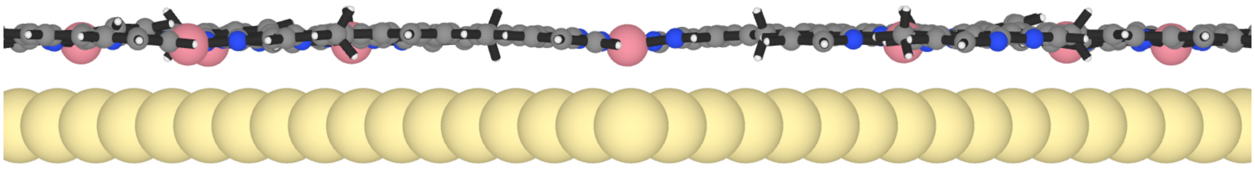

Figure S18: Side view of MOF absorbed on single layer of Au111 obtained from DFT-PBE optimized periodic structure. The Co atoms are ≈ 3.12Å above the Au(111).

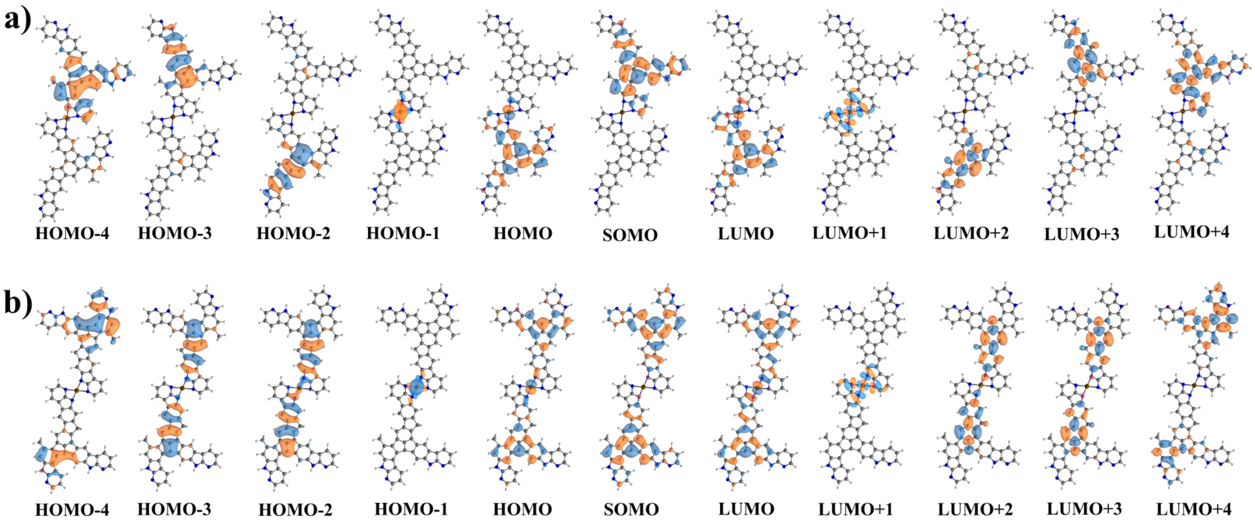

Figure S19: DFT unrestricted natural orbital (UNOs) used for the CASCI(11,11) for a) dimer having 1-2 coupling; b) dimer having 3-3 coupling.

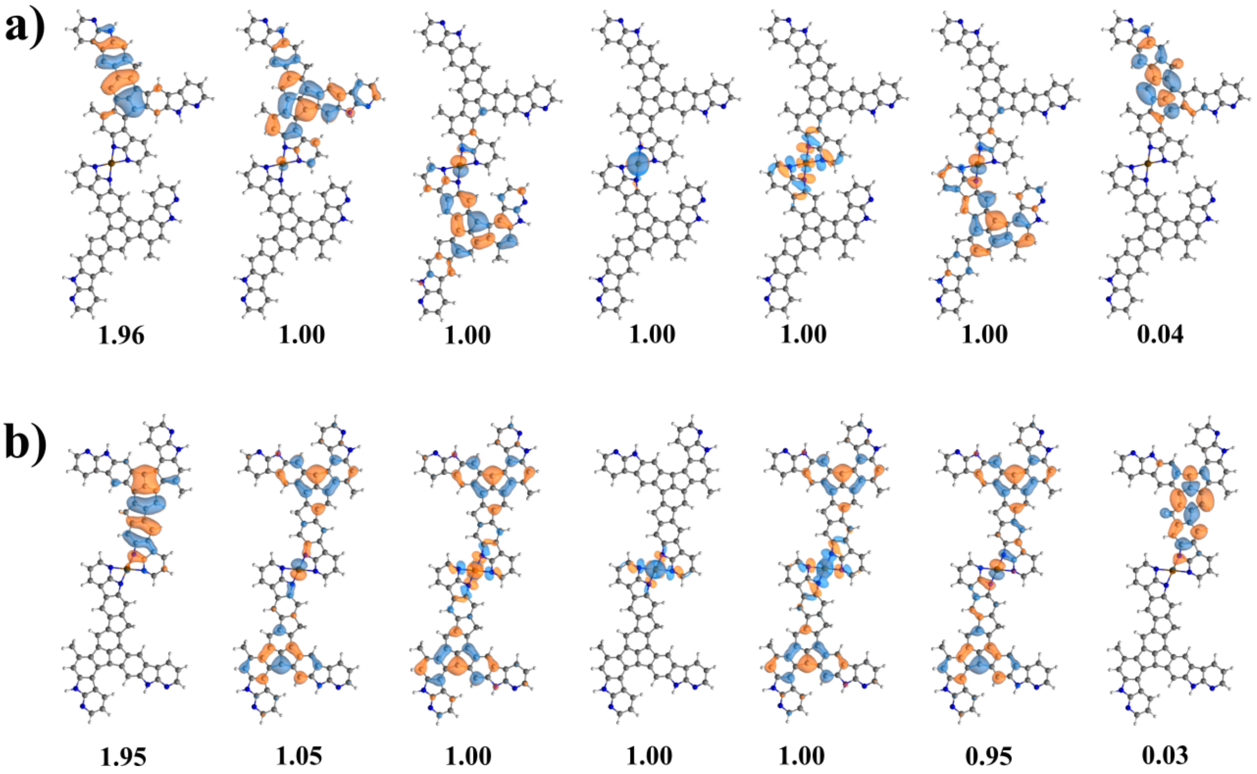

Figure S20: Natural orbital obtained from the CASCI calculations for a) dimer having 1-2 coupling; b) dimer having 3-3 coupling.

Table S1: Total energies, spin, and S^2 values for the first eight states for the dimers with 1-2 and 3-3 couplings. Active spaces used in these calculations are shown in Figure S19 (a) and (b), respectively.

State	Dimer 1-2			Dimer 3-3		
	Energy (mV)	Spin	S^2	Energy (mV)	Spin	S^2
0	0.0	2.5	8.75	0.0	0.5	0.75
1	7.9	1.5	3.75	4.2	1.5	3.75
2	12.3	1.5	3.75	11.5	2.5	8.75
3	13.2	0.5	0.75	49.8	1.5	3.75
4	328.0	1.5	3.75	398.4	0.5	0.75
5	333.2	0.5	0.75	416.6	1.5	3.75
6	339.6	0.5	0.75	453.2	0.5	0.75
7	531.0	1.5	3.75	559.6	1.5	3.75

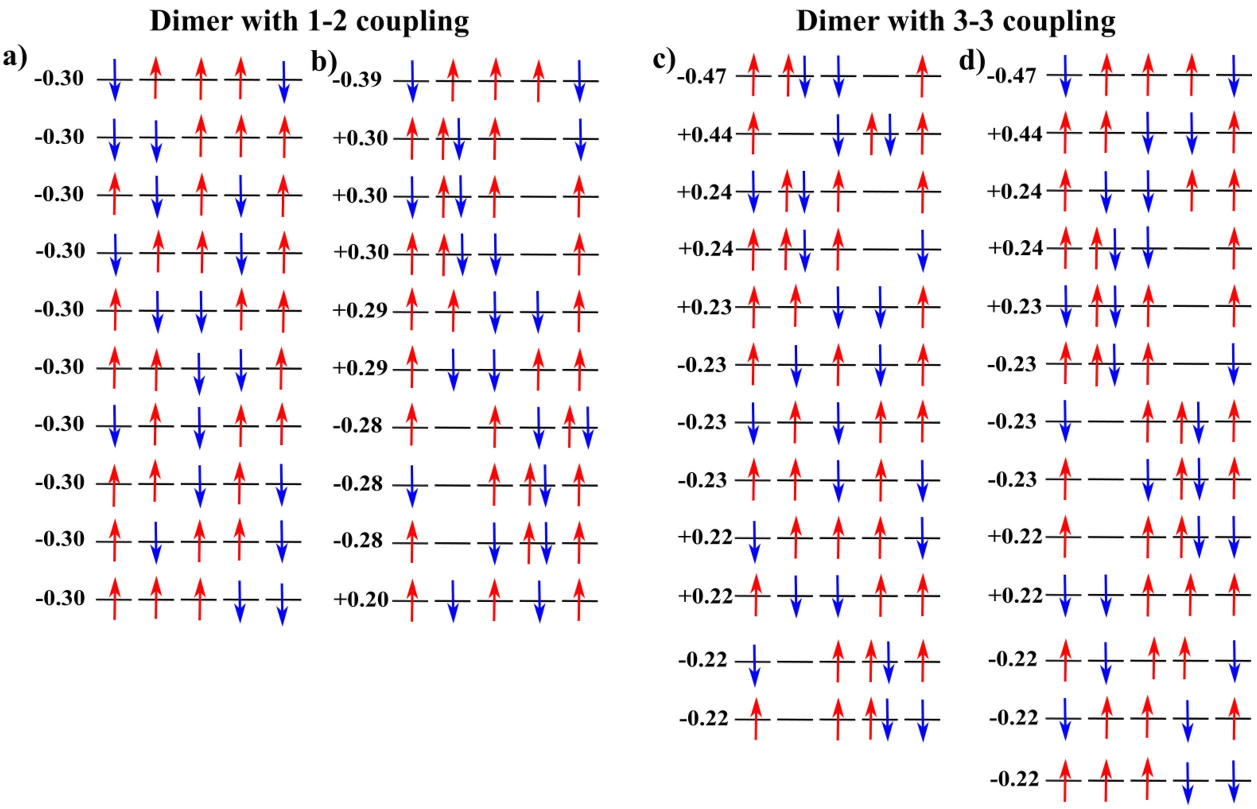

Figure S21: Many body wavefunctions for the dimer of 1-2 coupling a) the ground state b) the first excited state. c) The ground state of the dimer has 3-3 coupling and d) the first excited state of the dimer has 3-3 coupling. All the calculations are in the Sz subspace of 1. The MO basis set in representation are shown in Figure S19.

Table S2: Total energies, spin, and S2 value for the first eight states for trimer unit forming one sub-lattice. Active space used in this calculation is shown in Figure S23.

State	Energy (mV)	Spin	S^2
0	0.0	6	42
1	3.0	5	30
2	11.0	4	20
3	15.0	3	12
4	21.0	1	2
5	22.0	2	6
6	23.0	0	0
7	131.0	2	6

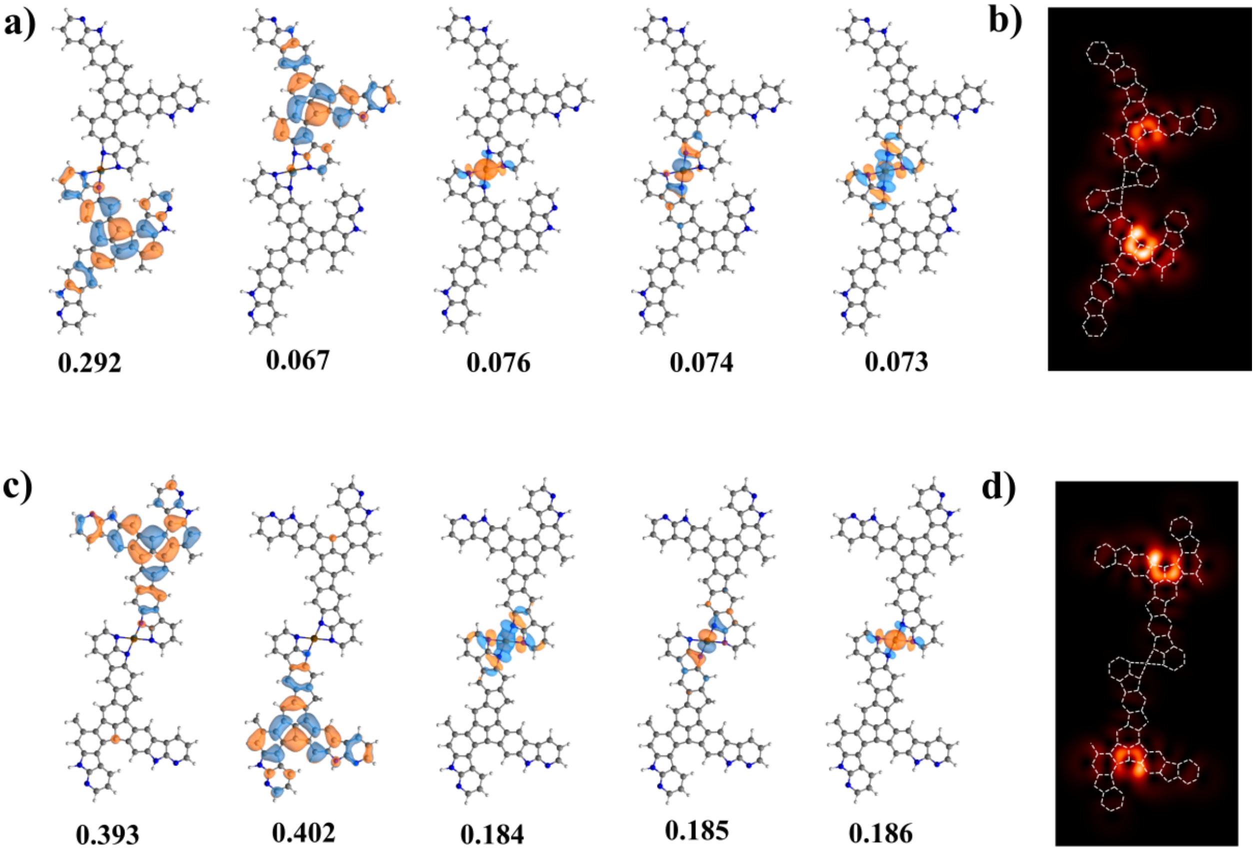

Figure S22: a) Natural transition orbitals with the non-zero weight for the transition from sextet to quartet for the dimer having 1-2 coupling; b) Simulated dI/dV maps corresponding to NTOs for dimer having 1-2 coupling using the PP-STM code[S13] for CO-like tip; c) Natural transition orbitals with the non-zero weight for the transition from sextet to quartet for the dimer having 3-3 coupling; d) Simulated dI/dV maps corresponding to NTOs for dimer having 3-3 coupling using the PP-STM code[S13] for CO-like tip.

Table S3: Total energy from the DFT (PBE0) with the different amounts of Hartree-Fock exchange for the trimer forming a sublattice.

HF xc%	Co=3↑, C=1↑	Co=1↑, C=1↑	Co=3↑, C=-1↓	Co=1↑, C=-1↓
25	1.198	0.000	1.231	0.014
35	0.482	0.000	0.519	0.000
45	0.000	0.008	0.041	0.007

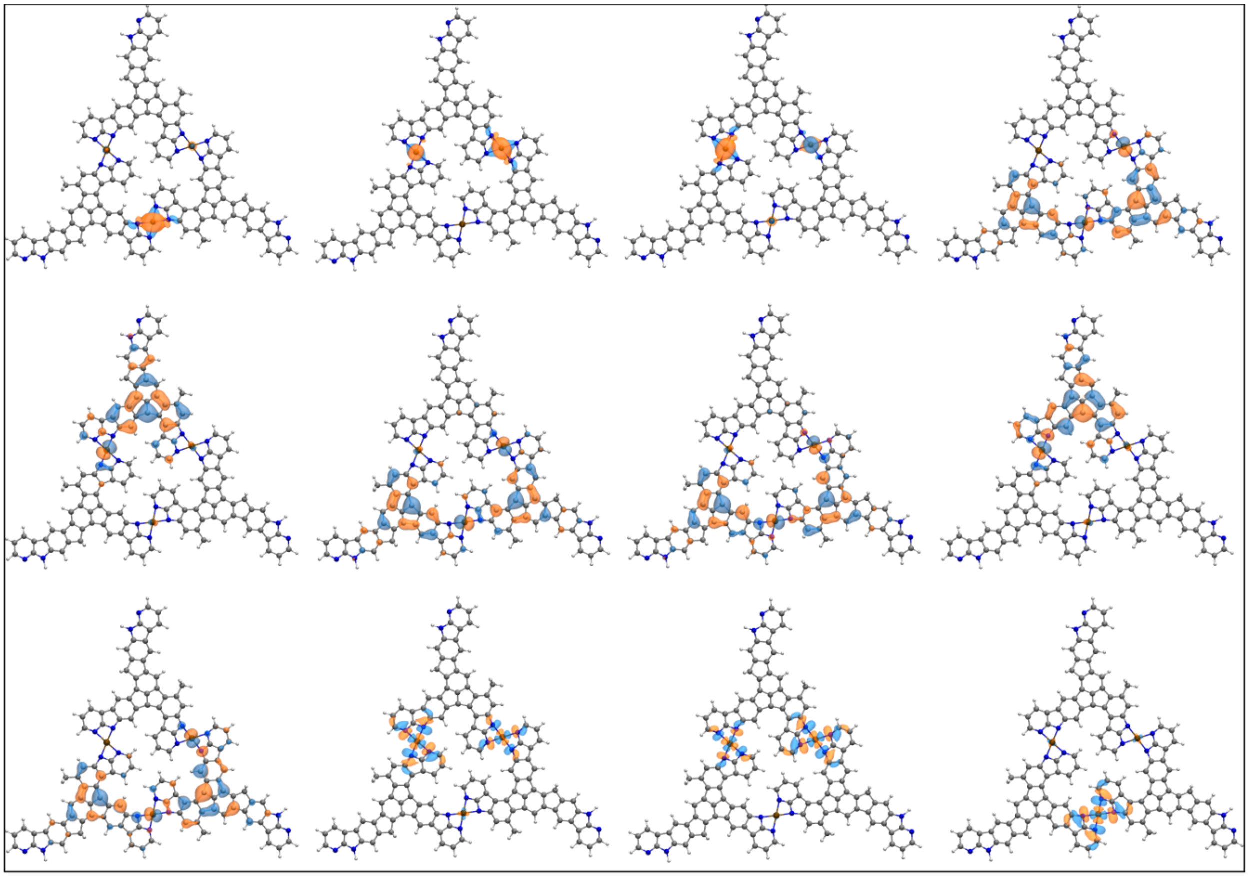

Figure S23: DFT unrestricted natural orbital (UNOs) used for the CASCI(12,12) for the trimer sublattice.

References

(S1) Frezza, F.; Matej, A.; Sánchez-Grande, A.; Carrera, M.; Mutombo, P.; Kumar, M.; Curiel, D.; Jelínek, P. On-Surface Synthesis of a Radical 2D Supramolecular Organic Framework. *Journal of the American Chemical Society* **2024**, *146*, 3531–3538.

(S2) Ormaza, M.; Bachellier, N.; Faraggi, M. N.; Verlhac, B.; Abufager, P.; Ohresser, P.; Joly, L.; Romeo, M.; Scheurer, F.; Bocquet, M.-L.; others Efficient spin-flip excitation of a nickelocene molecule. *Nano letters* **2017**, *17*, 1877–1882.

(S3) Horcas, I.; Fernández, R.; Gomez-Rodriguez, J.; Colchero, J.; Gómez-Herrero, J.; Baro, A. M. WSXM: A software for scanning probe microscopy and a tool for nanotechnology. *Review of scientific instruments* **2007**, *78*.

(S4) Blum, V.; Gehrke, R.; Hanke, F.; Havu, P.; Havu, V.; Ren, X.; Reuter, K.; Scheffler, M. Ab initio molecular simulations with numeric atom-centered orbitals. *Computer Physics Communications* **2009**, *180*, 2175–2196.

(S5) Perdew, J. P.; Burke, K.; Ernzerhof, M. Generalized Gradient Approximation Made Simple. *Phys. Rev. Lett.* **1996**, *77*, 3865–3868.

(S6) Tkatchenko, A.; Scheffler, M. Accurate Molecular Van Der Waals Interactions from Ground-State Electron Density and Free-Atom Reference Data. *Physical review letters* **2009**, *102*, 073005.

(S7) Perdew, J. P.; Ernzerhof, M.; Burke, K. Rationale for mixing exact exchange with density functional approximations. *The Journal of Chemical Physics* **1996**, *105*, 9982–9985.

(S8) Szabo, A.; Ostlund, N. S. *Modern quantum chemistry: introduction to advanced electronic structure theory*; Courier Corporation, 1996.

(S9) Neese, F. The ORCA program system. *WIREs Computational Molecular Science* **2012**, *2*, 73–78.

(S10) Martin, R. L. Natural transition orbitals. *The Journal of chemical physics* **2003**, *118*, 4775–4777.

(S11) Abad, J.; Martínez, J. I.; Gómez, P.; Más-Montoya, M.; Rodríguez, L.; Cossaro, A.; Verdini, A.; Floreano, L.; Martín-Gago, J. A.; Curiel, D.; others Two-Dimensional Self-Assembly Driven by Intermolecular Hydrogen Bonding in Benzodi-7-azaindole Molecules on Au (111). *The Journal of Physical Chemistry C* **2023**, *127*, 11591–11599.

(S12) Kanamori, J. Electron correlation and ferromagnetism of transition metals. *Progress of Theoretical Physics* **1963**, *30*, 275–289.

(S13) Krejčí, O.; Hapala, P.; Ondráček, M.; Jelínek, P. Principles and simulations of high-resolution STM imaging with a flexible tip apex. *Physical Review B* **2017**, *95*.
